# TCR ITAM multiplicity is required for the generation of follicular helper T-cells

**DOI:** 10.1038/ncomms7982

**Published:** 2015-05-11

**Authors:** SuJin Hwang, Amy C. Palin, LiQi Li, Ki-Duk Song, Jan Lee, Jasmin Herz, Noah Tubo, Hamlet Chu, Marion Pepper, Renaud Lesourne, Ekaterina Zvezdova, Julia Pinkhasov, Marc K. Jenkins, Dorian McGavern, Paul E. Love

**Affiliations:** 1 Program in Genomics of Differentiation, Eunice Kennedy Shriver National Institute of Child Health and Human Development, National Institutes of Health, Room 2B-210, Building 6B, Bethesda, Maryland 20892, USA; 2 Viral Immunology and Intravital Imaging Section, National Institute of Neurological Disorders and Stroke, National Institutes of Health, Bethesda, Maryland 20892, USA; 3 Department of Microbiology, Center for Immunology, University of Minnesota Medical School, Minneapolis, Minnesota 55455, USA

## Abstract

The T-cell antigen receptor (TCR) complex contains 10 copies of a di-tyrosine Immunoreceptor-Tyrosine-based-Activation-Motif (ITAM) that initiates TCR signalling by recruiting protein tyrosine kinases. ITAM multiplicity amplifies TCR signals, but the importance of this capability for T-cell responses remains undefined. Most TCR ITAMs (6 of 10) are contributed by the CD3ζ subunits. We generated ‘knock-in’ mice that express non-signalling CD3ζ chains in lieu of wild-type CD3ζ. Here we demonstrate that ITAM multiplicity is important for the development of innate-like T-cells and follicular helper T-cells, events that are known to require strong/sustained TCR–ligand interactions, but is not essential for ‘general’ T-cell responses including proliferation and cytokine production or for the generation of a diverse antigen-reactive TCR repertoire.

## Abstract (web summary)

The strength of TCR activation shapes T cell selection and differentiation. Here the authors show by modulating the number of activatory motifs in TCR complex that the strength of TCR signal is critical for the development of some T cell subsets but not for T cell effector functions or repertoire.

## Graphical abstract

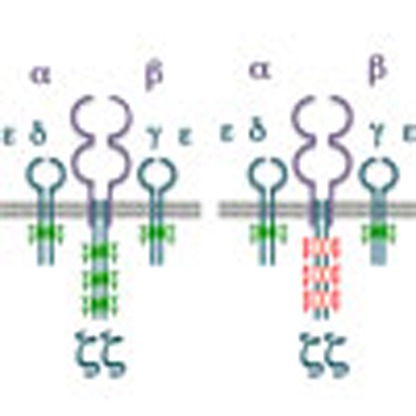

The T-cell antigen receptor (TCR) performs several essential cellular functions in T lymphocytes including conferring antigen recognition, initiation of activating signalling cascades in response to ligand binding, and regulation of key developmental steps required for intrathymic T-cell maturation. Subunits of the TCR complex can be classified into two distinct functional groups: ligand binding or signal transduction. The TCRs expressed by the majority of T lymphocytes contain clonotypic heterodimers composed of TCRα and TCRβ chain proteins that are generated by V(D)J recombination of germline gene segments during early stages of T-cell development in the thymus. TCRα/β dimers confer ligand-binding specificity and associate non-covalently with dimers composed of the invariant signal transducing subunits: CD3γ, CD3δ, CD3ε and CD3ζ. Although the exact subunit composition of mature αβTCR complexes has not been unequivocally established, current data support an octameric structure with the following stoichiometry: TCRαβ, CD3γε, CD3δε and CD3ζζ^1^,^2^.

Each of the invariant TCR subunits (CD3γ, CD3δ, CD3ε and CD3ζ) contains one or more copies of a semi-conserved sequence, the Immunoreceptor Tyrosine-based Activation Motif (ITAM), within their cytoplasmic domains that are composed of two YxxL/I cassettes (Y=tyrosine, L=leucine, I=isoleucine, x=any amino acid) separated by 6–8 amino acids^3^. ITAMs operate at the apex of the TCR signalling cascade and ITAM tyrosine phosphorylation is the earliest detectable signalling event that occurs following TCR cross-linking or ligand binding^4^. TCR engagement by peptide-MHC (pMHC) complexes results in membrane-dissociation of ITAMs and rapid phosphorylation of ITAM tyrosine residues by Src family protein tyrosine kinases^5^. Recruitment and activation of the dual SH2 domain protein tyrosine kinase ZAP-70 to tyrosine phosphorylated ITAMs promotes ZAP-70-mediated phosphorylation of the cytosolic adapters LAT and SLP-76, leading to the recruitment and activation of multiple effectors including Sos, PLCγ-1 and Vav1 that trigger Ras activation, calcium mobilization and cytoskeletal reorganization in T-cells, events that are essential for T-cell effector functions^6^,^7^. Although some TCR subunits contain additional conserved functional motifs, ITAMs are the predominant signal transducing sequences within the TCR complex^8^,^9^,^10^.

A longstanding and still unresolved question is why the TCR complex contains multiple signal transducing subunits and multiple ITAMs. CD3γ, CD3δ and CD3ε each contain one ITAM, whereas CD3ζ contains three ITAMs, yielding a total of 10 ITAMs within a single octameric TCR complex. Mutagenesis experiments in which individual ITAMs within the CD3 signalling subunits were inactivated have shown that no single ITAM is essential for either T-cell maturation or T-cell activation indicating that TCR ITAMs are at least partially functionally redundant^11^,^12^,^13^,^14^,^15^,^16^,^17^. Several groups have independently examined the importance of ITAM multiplicity for TCR-mediated signalling by generating mouse models where transgene or retrovirus encoded ITAM-mutant CD3ζ chains were expressed in CD3ζ^−/−^ mice^12^,^14^,^17^,^18^,^19^. Data from each study documented a requirement for CD3ζ ITAMs in regulating the set-point for both positive and negative thymocyte selection. However, the impact of reducing the TCR signalling potential on mature T-cell responses was not examined extensively, and the results obtained were inconsistent, likely reflecting differences in the experimental mouse models.

To address outstanding and unresolved questions concerning the role and importance of ITAM multiplicity for TCR-mediated signalling, we analysed two lines of ‘knock-in’ mice generated by gene targeting in embryonic stem cells: 6Y/6Y, which encodes a ‘wild-type’ CD3ζ chain, and 6F/6F, which encodes a CD3ζ protein where each of the 6 ITAM Y residues was mutated to Phenylalanine (F), rendering these ITAMs non-functional for signal transduction^20^ (Fig. 1a). Both ‘knock-in’ alleles were placed under the control of endogenous CD3ζ regulatory sequences so that expression of the 6Y and 6F CD3ζ proteins mimics that of endogenous CD3ζ, both developmentally and quantitatively^20^. In the current study, we used the 6Y/6Y and 6F/6F mouse models to investigate the importance CD3ζ ITAMs, and by extension, TCR ITAM multiplicity, for T-maturation and T-cell effector functions. Unexpectedly, we found that attenuation of the TCR signalling potential has an apparently negligible impact on generation of a broad antigen-reactive TCR repertoire or on ‘general’ T-cell responses such as proliferation and cytokine production. However, the maturation of innate-like T-cells (γδ T-cells and iNKT T-cells) as well as the generation of T follicular helper (TFH) cells, events that are known to depend on TCR interactions that result in long dwell times and high signal intensity, were markedly impaired in 6F/6F mice. These results reveal that a key function of ITAM multiplicity is to facilitate developmental and functional responses that are dependent on strong or sustained TCR/ligand interactions.

## Results

### TCR ITAM reduction results in altered TCR-Vα chain usage

**Figure 1:**
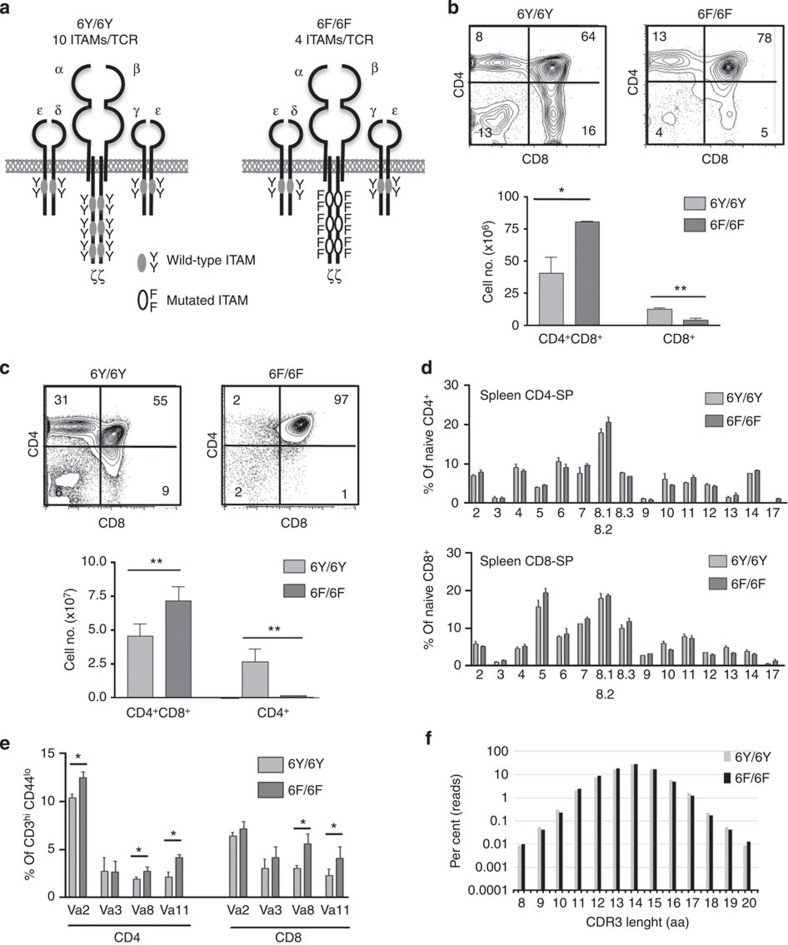
T-cell development in 6F/6F mice. (**a**) Schematic of αβTCR complexes expressed in 6Y/6Y and 6F/6F mice. Left, TCR complex expressed on αβ T-cells in 6Y/6Y mice containing a total of 10 ITAMs contributed by CD3γ(1) CD3δ(1) CD3ε(2) and CD3ζζ(6). Right, TCR complex expressed on αβ T-cells in 6F/6F mice containing a total of four ITAMs contributed by CD3γ(1) CD3δ(1) CD3ε(2). In 6F/6F mice, the three ITAMs within each TCRζ chain were inactivated by substitution of both Tyrosine (Y) residues within each ITAM with Phenylalanine (F) residues. (**b**) Upper panels, representative CD4 versus CD8 staining profiles of thymocytes from H-Y TCR transgenic female 6Y/6Y or 6F/6F mice. Lower panels, number of CD4^+^CD8^+^ and CD4^−^CD8^+^ (CD8^+^) thymocytes in H-Y TCR transgenic female 6Y/6Y or 6F/6F mice. *N*=13 mice of each genotype. (**c**) Upper panels, representative CD4 versus CD8 staining profiles of thymocytes from AND TCR transgenic 6Y/6Y or 6F/6F mice. Lower panels, number of CD4^+^CD8^+^ and CD4^+^CD8^−^ (CD4^+^) thymocytes in AND TCR transgenic 6Y/6Y or 6F/6F mice. *N*=10 mice of each genotype. (**d**) Vβ surface staining of naïve (CD44^lo^ CD25^−^) CD3^hi^ CD4-SP splenocytes (top) or naïve (CD44^lo^) CD3^hi^ CD8-SP splenocytes (bottom). Data are from 10 gender-matched 8-week-old mice of each genotype. (**e**) Vα surface staining of naïve (CD44^lo^ CD25^−^) CD3^hi^ CD4-SP splenocytes (left) or naive (CD44^lo^) CD3^hi^ CD8-SP splenocytes (right) from 6Y/6Y and 6F/6F mice. Data are from 10 gender-matched 8-week-old mice of each genotype. For **b**–**e**, data were analysed by unpaired *t*-test (two tailed) and are represented as mean±s.d. *P<0.05; **P<0.01. (**f**) CDR3 lengths (per cent of total in-frame reads) of TCR-Vβ loci from naïve CD4-SP T-cells from 6Y/6Y or 6F/6F mice.

The predicted subunit composition of αβTCRs expressed in homozygous germline 6Y/6Y and 6F/6F ‘knock-in’ mice is depicted in Fig. 1a. Since the mature TCR complex (TCRαβ/CD3γε/CD3δε/CD3ζζ) contains a total of 10 ITAM signalling motifs (6 contributed by the CD3ζζ homodimer and 4 contributed by the CD3γε and CD3δε dimers) the signalling potential of the TCR is theoretically reduced by 60% (6/10 ITAMs) in 6F/6F mice relative to ‘wild-type’ 6Y/6Y mice.

As demonstrated previously^20^ T-cell development is only mildly impaired in 6F/6F mice; the main defect being a reduction in the number of mature CD4^+^CD8^−^ (CD4-single positive (CD4-SP)) and CD4^−^CD8^+^ (CD8-SP) thymocytes and peripheral CD4-SP and CD8-SP T-cells relative to 6Y/6Y control mice. Based on our previous findings, we suspected that reduction of the TCR signalling potential might alter the repertoire of TCRs that successfully navigate the selection process in the thymus^20^. Indeed, positive selection of 6F/6F thymocytes expressing a transgenic MHC Class I (MHC I)-restricted (H-Y) or an MHC Class II (MHC II)-restricted (AND) αβTCR transgene was almost completely blocked at the immature CD4^+^CD8^+^ (double positive (DP)) stage (Fig. 1b,c). Moreover, CD8-SP thymocytes and T-cells in H-Y; 6F/6F mice and CD4-SP T-cells in AND; 6F/6F mice were almost exclusively T3.70^−^ or Va11^−^, respectively, indicating that their maturation required the expression of endogenously encoded TCRα chain(s) (Supplementary Fig. 1). We detected no significant difference in TCR-Vβ chain usage by polyclonal CD4-SP or CD8-SP thymocytes or naïve (CD44^lo^ CD62L^+^) CD4-SP or CD8-SP T-cells in 6Y/6Y and 6F/6F mice (Fig. 1d). However, TCR-Vα usage was significantly different in mature (CD24^lo^) CD4-SP and CD8-SP thymocytes and naïve (CD44^lo^) CD4-SP and CD8-SP peripheral T-cells from 6Y/6Y and 6F/6F mice (Fig. 1e and Supplementary Fig. 2a). Moreover, when TCRβ expression was fixed by introduction of a Vβ3 transgene, the frequency of Vβ3 pairing with its preferred Vα partners (Vα11 for CD4-SP and Vα8 for CD8-SP thymocytes and T-cells) was significantly different in 6Y/6Y and 6F/6F mice (Supplementary Fig. 2b).

To investigate the TCR-Vβ chain repertoire in greater detail, we performed next generation deep sequencing of rearranged TCRβ chain genes in purified naïve CD4-SP T-cells from 6Y/6Y, 6F/6F and C57BL/6 (B6) mice. CDR3 length distributions of productive TCRβ rearrangements (Fig. 1f) and CDR3 sequence diversity (Supplementary Fig. 2c) was similar in 6Y/6Y and 6F/6F samples. In addition, linear regression analysis demonstrated that the extent of CDR3 sequence identity between 6Y/6Y and 6F/6F CD4-SP T-cells was similar to that between 6Y/6Y and B6 CD4-SP T-cells (Supplementary Fig. 2c). Taken together, these results indicate that attenuation of TCR signalling potential does not prevent thymocyte positive selection and does not alter TCR-Vβ usage but instead results in skewing of the TCR repertoire mainly, if not entirely, through changes in TCRα chain usage.

### Defective clonal deletion in 6F/6F mice

In contrast to positive selection, which is mediated by relatively low affinity TCR–ligand interactions and low signal intensity TCR signalling, negative selection requires high intensity TCR signalling^21^,^22^. We previously demonstrated that negative selection is impaired in 6F/6F mice as assessed by a reduction in superantigen-mediated deletion; however, overt autoimmunity is not observed in 6F/6F mice^20^. In a recent report, it was show that in addition to clonal deletion, autoreactive thymocytes can also be negatively selected by ‘developmental diversion’ into αβTCR^+^ CD4^−^CD8^−^ (double negative (DN)) thymocytes^23^. Although clonal deletion normally predominates in wild-type mice, accumulation of developmentally diverted αβTCR^+^ DN thymocytes can be observed in the absence of CD28 co-stimulation or if apoptosis is prevented by overexpression of the pro-survival factors Bcl-2 or Mcl-1 (ref. 23). Examination of 6F/6F DN thymocytes revealed that reduction of TCR signalling potential results in an increase in ‘developmentally diverted’ CD5^hi^, PD-1^hi^ and CD69^+^ αβTCR^+^ DN thymocytes, even in the presence of CD28 co-stimulation (Supplementary Fig. 2d). The increase in developmentally diverted thymocytes in 6F/6F mice is presumably due to the fact that these cells have escaped clonal deletion as a result of reduced TCR signalling potential.

**Figure 2:**
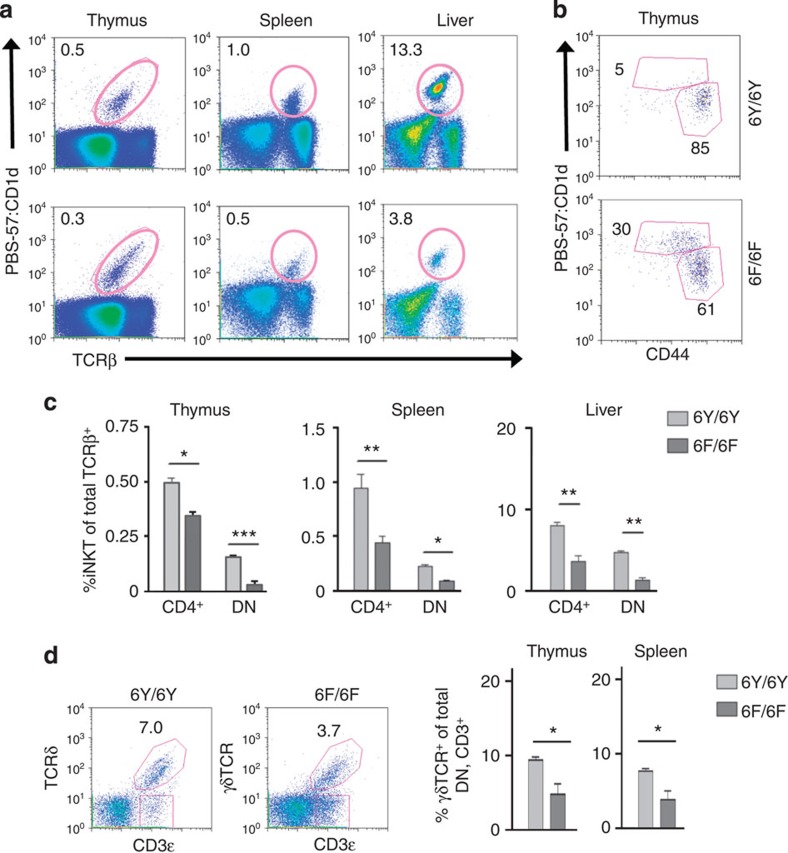
iNKT-cell and γδ T-cell development in 6Y/6Y and 6F/6F mice. (**a**) Representative (one of eight) PBS-57:CD1d-tetramer versus TCRβ profiles of thymocytes, splenocytes or lymphocyte-enriched cells from liver. (**b**) Representative (one of five) staining of CD4-SP thymocytes with PBS-57:CD1d-tetramer versus CD44. Percentages of TCRβ^+^ CD44^lo^ PBS-57:CD1d-tetramer^+^, and TCRβ^+^ CD44^hi^ PBS-57:CD4-CD1d-tetramer^+^, iNKT-cells within the indicated gates are shown. (**c**) Summary of PBS-57:CD1d-tetramer staining experiments. Shown are per cent PBS-57:CD1d-tetramer-positive cells within total thymocytes, total splenocytes or lymphocyte-enriched cells from liver. *N*=5 mice of each genotype. (**d**) Left panels, representative CD3ε versus TCRδ staining of CD4^−^CD8^−^ thymocytes from 6Y/6Y and 6F/6F mice. Right, bar graph shows summary data from six age-matched mice of each genotype. For **c**,**d**, data were analysed by unpaired *t*-test (two tailed) and are represented as mean±s.d. *P<0.05; **P<0.01.

High intensity TCR signalling is also required for maturation of innate-like T-cells, which include iNKT-cells and γδTCR^+^ T-cells (γδ T-cells) that express invariant or semi-invariant TCRs and are thought to encounter their cognate antigens in the thymus^24^,^25^,^26^,^27^. We observed a significant reduction in the number of PBS-57:CD1d–tetramer^+^ thymocytes and peripheral iNKT-cells in 6F/6F mice (Fig. 2a–c). Consistent with the results of a previous study^28^, a higher percentage of PBS-57:CD1d tetramer^+^ CD4-SP thymocytes in 6F/6F mice were immature (CD44^lo^, TCR^hi^) indicative of a block in iNKT-cell development^29^ (Fig. 2b). We also detected a significant reduction of γδTCR^+^ thymocytes and peripheral γδ T-cells in 6F/6F mice (Fig. 2d). These findings demonstrate that a full complement of TCR ITAMs is required for the generation of T-cell populations that express invariant TCRs and that are known to require relatively high affinity/high intensity intrathymic signalling for their complete maturation.

### Signalling responses are unequally impacted in 6F/6F mice

**Figure 3:**
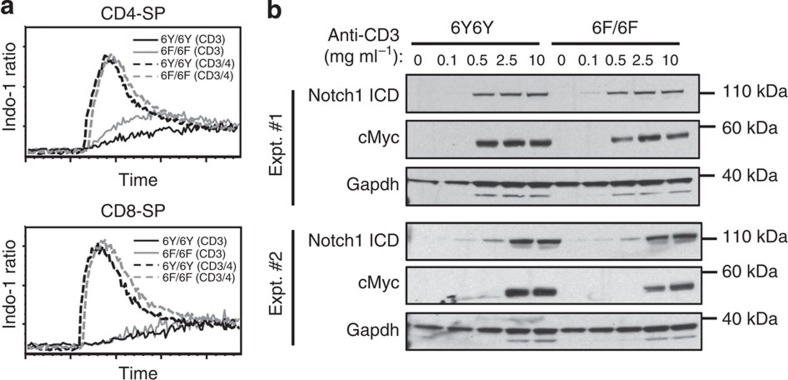
TCR-mediated signalling responses in 6F/6F mice. (**a**) Calcium flux in naïve (CD44^lo^ CD25^−^) 6Y/6Y and 6F/6F CD4-SP T cells (upper panel) or naïve (CD44^lo^) CD8-SP T-cells (lower panel) induced by stimulation with the indicated antibodies (time frame, 5 min). One representative of three independent experiments. (**b**) Induction of Notch ICD and c-Myc in 6Y/6Y and 6F/6F T-cells. Naïve (CD44^−^ CD25^−^) CD4-SP T-cells were stimulated for 24 h on plates coated with anti-CD28 (50 μg ml^−1^) and the indicated amount of anti-CD3. Whole cell extracts were analysed by SDS–PAGE and Western blotting with the indicated antibodies.

Analysis of signalling pathways downstream of the TCR revealed that, similar to what we had previously found in thymocytes^20^, activation of TCR-proximal signalling effectors including ZAP-70, SLP-76 and Cbl was reduced in 6F/6F peripheral T-cells relative to 6Y/6Y T-cells under the same stimulation conditions (Supplementary Fig. 3a,b). However, the effect of reducing TCR signalling potential on activation of more distal effectors and responses was variable. For example, calcium mobilization was unaffected in 6F/6F T-cells, whereas Akt and Erk activation were strongly attenuated (Fig. 3a, Supplementary Fig. 3a,b). In contrast to the results obtained with a different CD3ζ ITAM-mutant model system^19^, we found that CD3ζ ITAMs were not critical for either c-Myc or Notch activation in response to TCR engagement (Fig. 3b). From these results, we conclude that loss of CD3ζ-chain signalling does not equivalently impact the activation of signalling effectors downstream of the TCR, suggesting that there may be different thresholds for the activation of individual signalling intermediates and pathways.

### T-cell proliferation is minimally impacted in 6F/6F mice

**Figure 4:**
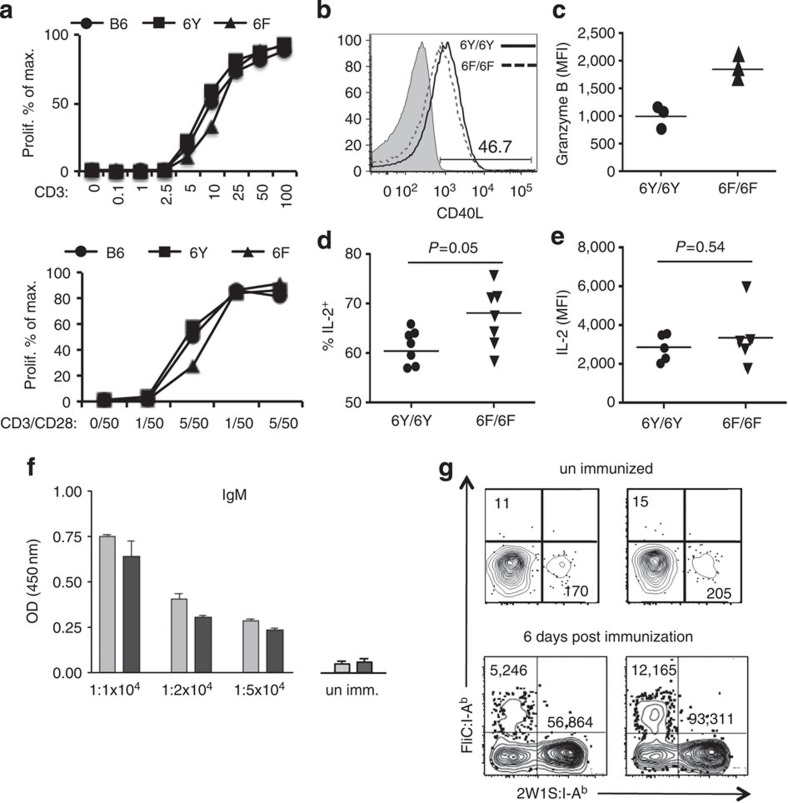
T-cell functional responses in 6F/6F mice. (**a**) Naïve (CD44^lo^ CD25^−^) CD4-SP lymph node T-cells were pre-loaded with CFSE and stimulated for 3 days with the indicated amount of plate-bound anti-CD3 antibody (top) or anti-CD3+anti-CD28 antibodies (bottom). Results are shown as per cent of maximum proliferation with PMA+ionomycin as assessed by flow cytometric analysis of CFSE dilution. One representative of three independent experiments. (**b**) Naïve CD4-SP T-cells were stimulated with plate-bound anti-CD3+anti-CD28 antibodies overnight then examined for CD40L expression by surface staining and flow cytometry. (**c**) Naïve CD8-SP T-cells were stimulated with plate-bound anti-CD3+anti-CD28 antibodies for 3 days, incubated with IL-2 (100 U ml^−1^) for 24 h, restimulated with PMA+ionomycin for 4 h, then examined for granzyme B expression by intracellular staining and flow cytometry. (**d**,**e**) Naïve CD4-SP T-cells were stimulated with plate-bound anti-CD3+anti-CD28 for 2 days and the percentage of IL-2^+^ cells (**d**) and the MFI of IL-2 (**e**) was determined by intracellular staining. (**f**) IgM antibody response to T-dependent antigens. Mice were immunized with NP-KLH in CFA. One week after immunization, sera obtained from tail bleeds were analysed for NP-reactive IgM by enzyme-linked immunosorbent assay. Twelve mice were analysed in each group. (**g**) Enumeration of antigen-specific T-cell pools in 6F/6F mice. Total numbers of lymph node and spleen 2W/I-A^b^-specific and FliC/I-A^b^-specific CD4-SP T-cells in 6Y/6Y (left panels) and 6F/6F (right panels) mice were analysed by tetramer enrichment. Upper panels, number of peptide-specific CD4-SP peripheral T-cells in un-immunized mice. Lower panels, number of peptide-specific CD4-SP peripheral T-cells in mice 6 days after i.v. immunization with peptide+LPS. Shown are one representative of two to four independent experiments.

To determine if reducing the number of TCR ITAMs impairs mature T-cell functional responses, we first analysed *in vitro* TCR antibody-mediated proliferation and cytokine production. Notably, naïve CD4-SP and CD8-SP T-cells from 6F/6F mice exhibited only mildly impaired proliferative responses to either anti-CD3 induced stimulation or stimulation with anti-CD3 plus anti-CD28 (Fig. 4a, Supplementary Fig. 4a). We consistently observed a slight reduction in the proliferative response of 6F/6F T-cells with lower amounts of stimulating antibody, but proliferation was equivalent to that of 6Y/6Y T-cells when the amount of activating antibody was increased. Induction of CD69 and the interleukin-2Rα (IL-2Rα) chain (CD25) in response to TCR stimulation was also unaffected or only minimally impaired in 6F/6F T-cells (Supplementary Fig. 4b,c). Polyclonal 6F/6F CD4-SP and CD8-SP T-cells upregulated CD40L or granzyme B, respectively, in response to TCR cross-linking (Fig. 4b,c). A higher percentage of naïve 6F/6F T-cells produced IL-2 in response to TCR+CD28 stimulation although the total amount of IL-2 per cell was not increased (Fig. 4d,e). Interferon-γ (IFNγ) production by *in vitro*-activated naïve CD4-SP 6F/6F T-cells was also the same or greater than in 6Y/6Y T-cells, whereas IL-4 production by activated naïve 6F/6F CD4-SP T-cells was consistently reduced compared with identically treated naïve 6Y/6Y CD4-SP T-cells (Supplementary Fig. 4d,e). As reported previously^20^, 6F/6F mice are lymphopenic and contain increased percentages of CD44^hi^ CD62L^lo^ ‘memory-phenotype’ CD4-SP and CD8-SP T-cells (Supplementary Fig. 5a). We observed that a higher percentage of *ex vivo* CD44^hi^ CD62L^lo^ CD4-SP and CD8-SP T-cells in 6F/6F mice produced IL-2, IFNγ and TNFα as assessed by intracellular staining (Supplementary Fig. 5b).

### No overt gaps in the 6F/6F antigen-reactive TCR repertoire

The differences in TCRα chain usage by positively selected 6F/6F T-cells relative to control 6Y/6Y T-cells raised the question of whether there are ‘gaps’ in the antigen-reactive T-cell repertoire in 6F/6F mice that would manifest by failure to respond to particular foreign antigens. To investigate this, we first immunized mice with the T-dependent antigen NP-Keyhole limpet haemocyanin (NP-KLH). As shown in Fig. 4f, 6F/6F mice mounted NP IgM antibody responses to NP-KLH that were similar to those of 6Y/6Y control mice. Similar results were obtained with another T-dependent antigen, NP-chicken gamma-globulin (NP-CGG).

Next, we determined the total number of naïve T-cells that are specific for the I-A^b^ MHC II-binding non-self-peptides, 2W, a variant of peptide 52–68 from the I-E alpha chain, and FliC, peptide 427–441 from the FliC protein of *Salmonella typhimurium*, or the K^b^ MHC I-binding peptide from ovalbumin (OVA 257–264). Peptide-specific T-cells from spleen and total lymph nodes from each mouse were enriched with the relevant pMHC tetramers as described^30^ and enumerated by flow cytometry. 6F/6F mice contained naïve 2 W-specific and FliC-specific CD4-SP T-cells as well as naïve OVA-specific CD8-SP T-cells that were present in numbers similar to those detected in 6Y/6Y mice (Fig. 4g and Supplementary Fig. 5c). We also detected peptide-specific CD4-SP and CD8-SP thymocytes in 6F/6F mice by tetramer enrichment (Supplementary Fig. 5c). To evaluate the proliferative potential of the naïve CD4-SP T-cell populations, mice were injected intravenously with 2W and FliC peptides plus lipopolysaccharide to elicit a synchronous systemic antigen-specific T-cell response. Six days later, total spleen and lymph node peptide-specific CD4-SP T-cells were tetramer enriched and enumerated. As shown in Fig. 4g, proliferative expansion of naïve 2W- and FliC-specific T-cells in 6F/6F mice was as good or better than that observed in 6Y/6Y mice. Therefore, the number and expansion potential of naïve T-cells specific for these epitopes was not decreased in 6F/6F mice.

**Figure 5:**
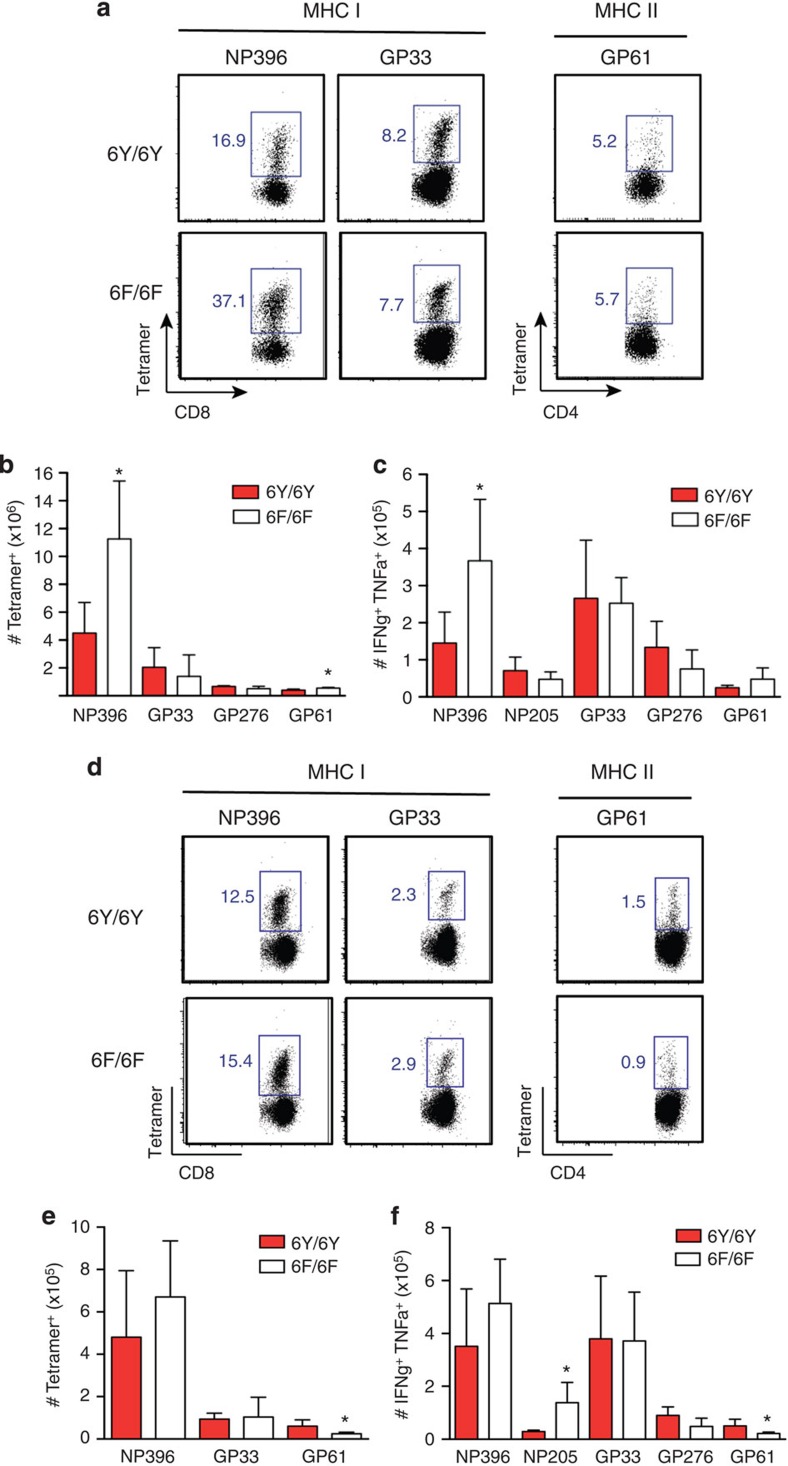
Generation of effector and memory T-cells in 6F/6F mice. (**a**–**c**) Mice were infected with LCMV Armstrong and spleen T-cells were analysed 8 days later. (**a**) Dot plots show percentage of tetramer-positive CD8-SP or CD4-SP splenocytes. (**b**) Number of tetramer-positive CD8-SP (NP396, GP33 and GP276) or CD4-SP (GP61) splenocytes in 6Y/6Y or 6F/6F mice. (**c**) Number of IFNγ^+^ TNFα^+^ splenocytes detected after 5 h *in vitro* re-stimulation with the indicated peptides. *N*=6 6Y/6Y and 6 6F/6F mice. Data were analysed by unpaired *t*-test (two tailed) and are represented as mean±s.e.m. *P<0.05. (**d**–**f**) Mice were infected with LCMV Armstrong and spleen T-cells were analysed 45 days after infection. (**d**) Dot plots show percentage of tetramer-positive CD8-SP or CD4-SP splenocytes. (**e**) Number of tetramer-positive CD8-SP or CD4-SP splenocytes in 6Y/6Y or 6F/6F mice. (**f**) Number of IFNγ^+^ TNFα^+^ splenocytes detected in 6Y/6Y or 6F/6F mice after 5 h *in vitro* re-stimulation with the indicated peptides. *N*=5 6Y/6Y and 6 6F/6F mice. Data were analysed by unpaired *t*-test (two tailed) and are represented as mean±s.e.m. *P<0.05.

We also infected age-matched 6Y/6Y and 6F/6F mice with LCMV Armstrong and evaluated LCMV antigen-specific responses 8 days after infection. To identify virus-specific T-cells, splenocytes from LCMV-infected mice were stained with MHC I or MHC II tetramers bound to individual LCMV-derived peptides. 6F/6F mice mounted T-cell responses to each of the LCMV-specific antigens that were screened by tetramer staining (Fig. 5a,b). Moreover some LCMV peptide-specific T-cell responses (for example, MHC I-restricted NP396–404 (NP396) and MHC II-restricted GP61–80 (GP61)) were significantly better (as assessed by the number of tetramer^+^ spleen T-cells detected 8 days after LCMV infection) in 6F/6F mice than in 6Y/6Y mice (Fig. 5a,b). Following *in vitro* re-stimulation with peptide, the number and percentage of peptide-specific IFNγ^+^TNFα^+^ T-cells paralleled tetramer binding, indicating that 6F/6F T-cells are capable of acquiring a *bona fide* effector phenotype (Fig. 5c and Supplementary Fig. 6a).

### Generation of memory T-cells is unimpaired in 6F/6F mice

We next evaluated the potential of 6F/6F T-cells to generate antigen-specific memory T-cells after LCMV Armstrong infection. Forty-five days after LCMV infection, memory T-cells reactive against each of the LCMV antigens that were screened by tetramer binding were detected in 6F/6F mice (Fig. 5d–f and Supplementary Fig. 6b). In general, memory T-cells in 6F/6F mice were composed of a higher percentage of CD62L^−^CD44^+^ T effector memory (T_EM_) and fewer CD62L^+^CD44^+^ central memory (T_CM_) phenotype cells compared with 6Y/6Y controls (Supplementary Fig. 7a). In accord with this finding, a higher percentage of tetramer-binding T-cells in 6F/6F mice, particularly GP61-reactive T-cells, expressed elevated levels of KLRG1^31^, (Supplementary Fig. 7b).

### Defective generation of TFH cells in 6F/6F mice

**Figure 6:**
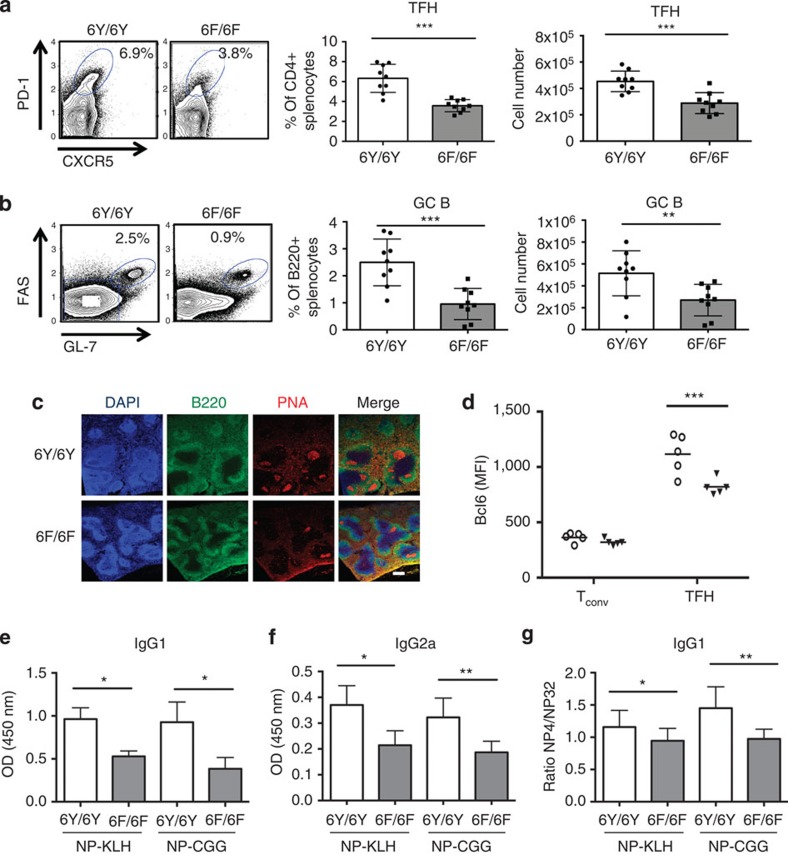
Impaired induction of T follicular helper (TFH) cells in 6F/6F mice. 6Y/6Y and 6F/6F mice were injected with sheep red blood cells (SRBCS) and 10 days later spleens were harvested for analysis. Percentage and numbers of (**a**) TFH (CD4^+^ CXCR5^+^ PD-1^+^) cells and (**b**) germinal centre B (GC B) cells (B220^+^ Fas^+^ GL-7^+^) detected in 6Y/6Y and 6F/6F mice. *N*=9 6Y/6Y and 9 6F/6F mice. (**c**) Immunofluorescence staining of spleens from 6Y/6Y and 6F/6F mice 1 week after immunization with SRBCS. One representative of three experiments. Scale bar, 200 μm. (**d**) Mean fluorescence intensity of anti-Bcl6 mAb staining of conventional CD4-SP T-cells (CD4^+^ CXCR5^−^ PD-1^−^) and TFH cells (CD4^+^ CXCR5^+^ PD-1^+^). (**e**–**g**) 6Y/6Y and 6F/6F mice (six each) were immunized with NP-KLH or NP-CGG. One week after immunization, serum was obtained by tail bleeding and analysed for NP-reactive IgG by enzyme-linked immunosorbent assay (ELISA). (**e**) Anti-NP IgG1. (**f**) Anti-NP IgG2a. (**g**) Ratio of high affinity/low affinity anti-NP IgG1 determined by ELISA with plates coated with NP(4) or NP(32). For bar graphs, data were analysed by unpaired *t*-test (two tailed) and are represented as mean±s.d. *P<0.05; **P<0.01, ***P<0.001.

Previous data have shown that strong TCR/pMHC interactions, particularly those with a long dwell time, favour the conversion of naïve CD4 T-cells into TFH^32^. Although this model predicts that the generation of TFH cells should also be dependent on stronger or more sustained TCR signals, a requirement for high intensity signalling has not been demonstrated experimentally. To evaluate TFH-cell development, age-matched 6Y/6Y and 6F/6F mice were injected with sheep red blood cells (SRBC), and 7–10 days later splenocytes were harvested and analysed by flow cytometry. As shown in Fig. 6a, the percentage and total number of TFH (CXCR5^+^ PD-1^+^) CD4-SP T-cells was significantly reduced in 6F/6F mice compared with 6Y/6Y controls. The reduction in TFH cells was not due to a delayed development as a similar reduction in TFH numbers was also detected in 6F/6F 14 days after immunization. In addition to their numerical reduction, TFH cells in 6F/6F mice expressed reduced levels of the transcription factor Bcl6 (Fig. 6d). Consistent with impaired generation of TFH cells, 6F/6F spleens contained fewer germinal centres (GCs) and reduced numbers of (Fas^+^ GL-7^+^) GC B-cells (Fig. 6b,c). A significant reduction in TFH cells and GC B-cells was also observed when 6F/6F mice were immunized with NP-CGG or NP-KLH. Analysis of NP-specific antibodies in immunized mice revealed impaired immunoglobulin class switching (Fig. 6e,f) and immunoglobulin affinity maturation (Fig. 6g) in 6F/6F mice also reflecting a defect in GC formation.

**Figure 7:**
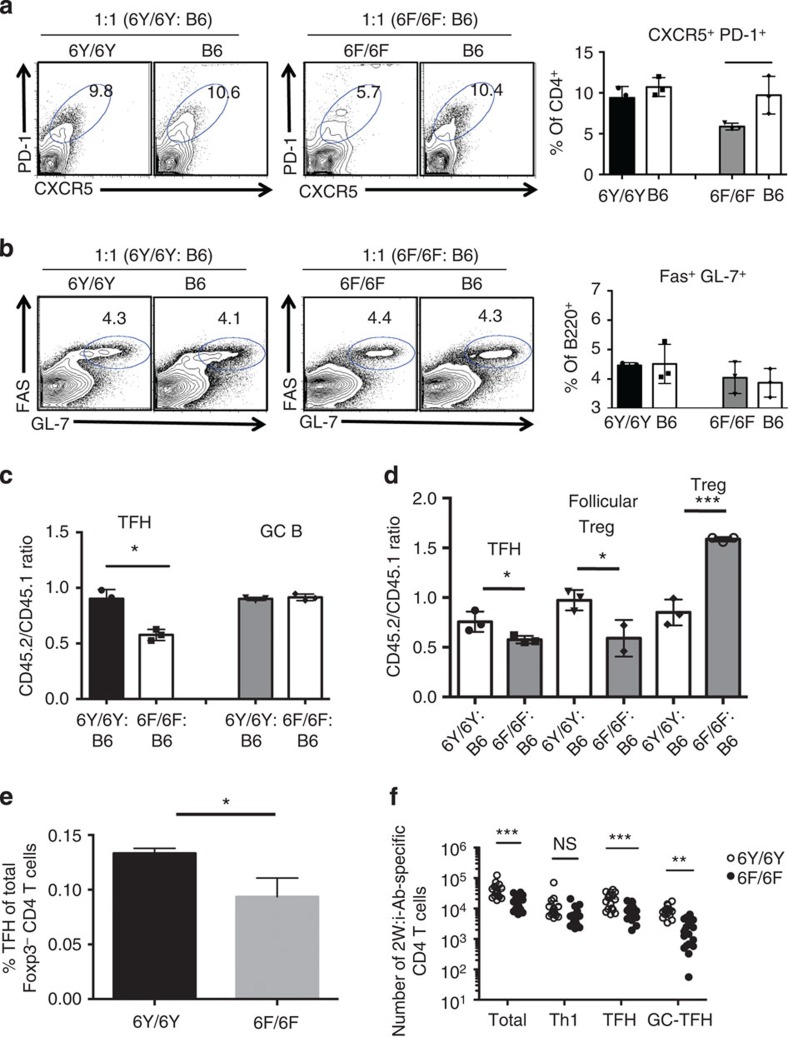
The defect in TFH induction in 6F/6F mice is T-cell intrinsic. (**a**–**e**) Bone marrow chimeras were generated by injecting a 1:1 mix of lineage-depleted 6Y/6Y and B6:CD45.1 cells or a 1:1 mix of lineage-depleted 6F/6F and B6.CD45.1 bone marrow cells into three lethally irradiated B6:CD45.1 mice. Six weeks later, mice were injected with sheep red blood cells (SRBC) and splenocytes were analysed 10 days after SRBC injection. (**a**) Dot plots show representative CXCR5 versus PD-1 staining of CD45.1-negative (6Y/6Y or 6F/6F) or CD45.1-positive (B6) CD4-SP splenocytes from 1:1 6Y/6Y:B6 or 1:1 6F/6F:B6 bone marrow chimeras. Bar graphs on right show results from all chimeric mice (3 6Y/6Y:B6 chimeras and 3 6F/6F:B6 chimeras). (**b**) Dot plots show representative Fas versus GL-7 staining of CD45.1-negative (6Y/6Y or 6F/6F) or CD45.1-positive (B6) B220^+^ splenocytes from 1:1 6Y/6Y:B6 or 1:1 6F/6F:B6 bone marrow chimeras. Bar graphs on right show results from all chimeric mice (three 6Y/6Y:B6 chimeras and three 6F/6F:B6 chimeras). (**c**) CD45.2/CD54.1 ratio of TFH and GC B-cells in 6Y/6Y:B6 and 6F/6F:B6 chimeras. (**d**) CD45.2/CD54.1 ratio of TFH (CD4^+^ CXCR5^+^ PD-1^+^), follicular Treg (CD4^+^ CXCR5^+^ PD-1^+^ Foxp3^+^) and Tregs (CD4^+^ Foxp3^+^) in 6Y/6Y:B6 and 6F/6F:B6 chimeras. (**e**) Percentage of TFH cells within all CD4^+^ Foxp3^−^ splenocytes. (**f**) Age- and gender- matched 6Y/6Y and 6F/6F mice (14 of each) were infected with *Listeria monocytogenes* that expresses 2W peptide, and splenocytes were analysed by flow cytometry 10 days after infection. The number of 2W:I-Ab-specific splenocytes was determined by tetramer enrichment. Th1 (CD4^+^IFNγ^+^), TFH (CD4^+^ CXCR5^+^PD-1^+^), GC-TFH (CD4^+^CXCR5^hi^ PD-1^hi^). For bar graphs, data were analysed by unpaired *t*-test (two tailed) and are represented as mean±s.d. *P<0.05; ***P<0.001.

Since Tregs are increased in 6F/6F mice^20^, we considered the possibility that this could explain the impaired TFH-cell response. To resolve this issue, we generated bone marrow chimeras by injecting lineage-depleted bone marrow cells from 6Y/6Y or 6F/6F mice (both CD45.2) together with an equal number of B6:CD45.1 lineage-depleted bone marrow cells into lethally irradiated B6:CD45.1 mice. Six weeks after bone marrow transfer, chimeric mice were immunized with SRBC and spleens were analysed 10 days later. As shown in Fig. 7a–c, a specific reduction in the development of 6F/6F TFH cells was observed in chimeric mice, demonstrating that the 6F/6F defect is T-cell intrinsic. In agreement with this conclusion, *in vitro* induction of CXCR5^+^ PD-1^+^ TFH-phenotype T-cells from naïve 6F/6F CD4-SP precursors was also impaired relative to 6Y/6Y controls (Supplementary Fig. 8a,b). 6F/6F T-cells were also defective in the generation of Foxp3^+^ CXCR5^+^ PD-1^+^ ‘Follicular Tregs’, whereas the generation of conventional Tregs was enhanced as previously reported^20^ (Fig. 7d). The reduction in 6F/6F TFH cells could not be attributed to a reduction in naïve CD4-SP precursors since TFH induction frequency was also significantly reduced compared with 6Y/6Y mice when calculated as per cent of total Foxp3^−^ cells (Fig. 7e).

Finally, we considered the possibility that the defect in generation of TFH cells in 6F/6F mice might be due to differences in the naïve antigen-specific T-cell pool as a result of effects of the 6F mutation on positive selection. To address this issue, we infected 6Y/6Y and 6F/6F mice with *Listeria monocytogenes* (Lm) that expresses a 2W peptide-OVA fusion protein (Lm-OVA) since we had established that 6Y/6Y and 6F/6F mice contain similar numbers of naïve 2W peptide-reactive CD4 T-cells (Fig. 4g). One week after infection, spleens were harvested and 2W tetramer-positive T-cells were enumerated by flow cytometry. As shown in Fig. 7f, splenocytes from 6F/6F mice contained significantly fewer numbers of 2W-reactive TFH as well as significantly fewer 2W-reactive (CXCR5^hi^ PD-1^hi^) GC-TFH cells relative to 6Y/6Y controls. The number of listeriolysin O peptide (LLO) tetramer-positive TFH cells and GC-TFH cells was also reduced in Lm-OVA-infected 6F/6F mice (Supplementary Fig. 8c). In contrast, the number of 2W-specific and LLO-specific IFNγ^+^ (Th1) CD4-SP T-cells was not significantly different in Lm-OVA-infected 6Y/6Y and 6F/6F mice, confirming a selective defect in the generation of TFH cells (Fig. 7f, Supplementary Fig. 8c).

## Discussion

More than two decades ago, the question of why the TCR contains (and presumably requires) multiple signal transducing subunits was first raised following initial reports of the multimeric subunit composition of the TCR. This query intensified on the discovery that TCR complexes contain as many as 10 copies of a semi-conserved (ITAM) signalling motif within their CD3 subunits. Despite continued investigation in the interim, a cogent resolution of the functional importance of TCR ITAM multiplicity has remained elusive, due in part to the finding that individual CD3 ITAMs are not assigned to couple the TCR to specific signalling pathways, and consequently are not indispensible for normal TCR signalling responses. Uncertainty regarding the purpose of TCR ITAM multiplicity can also be attributed in part to the inconsistent results obtained with different genetic reconstitution mouse models where the CD3ζ ITAMs were inactivated^12^,^14^,^17^,^18^,^19^. Each model used heterologous gene-expression methods to reconstitute expression of wild-type or mutant TCR signalling subunits in CD3ζ^−/−^ mice raising concerns that such non-physiological approaches might lead to confounding results, particularly in light of accumulated data revealing the complexity of the T-cell maturation process and its dependence on the precisely orchestrated expression of developmentally relevant genes.

For this study, we used a gene ‘knock-in’ mouse model designed to place expression of ‘wild-type’ (6Y) CD3ζ chain or a non-signalling tyrosine to phenylalanine-mutated (6F) CD3ζ chain cassette under the control of the endogenous *Cd3*ζ gene regulatory sequences. In a previous report, we demonstrated that expression of the 6Y and 6F ‘knock-in’ alleles faithfully mimics that of endogenous *CD3*ζ^20^. Here we document that αβ T-cell development is only mildly impaired in 6F/6F mice despite a theoretical 60% reduction in the signal transducing potential of the TCR. However, when the specificity of the TCR was fixed by introduction of an αβTCR transgene, positive selection of 6F/6F thymocytes expressing the defined TCR was almost completely abrogated. The T-cells that were generated in these mice were dependent on expression of rearranged endogenous TCRα chains to complete their maturation in the thymus. From these results, we infer that most αβTCRs that normally promote positive selection fail to transmit signals appropriate for positive selection in 6F/6F mice. Consequently, a different TCR repertoire is positively selected in 6F/6F mice. Our data also indicate that at least some of the TCR α/β heterodimers that are positively selected in 6F/6F mice bind with higher affinity to selecting self-ligands than most αβTCRs that normally promote positive selection as evinced by the increased percentage of ‘memory-phenotype’ peripheral T-cells in 6F/6F mice even in a lympho-replete background^20^.

An unexpected finding in the current study was that despite the profound effects of TCR ITAM reduction on positive selection, the extent of TCR-Vβ CDR3 diversity in the pool of naïve CD4-SP T-cells from 6Y/6Y and 6F/6F mice is no greater than it is between 6Y/6Y and B6 mice. Thus, the differences in the αβTCR repertoire in 6Y/6Y and 6F/6F mice can primarily be attributed to differences in TCRα chain usage by a similar repertoire of TCRβ chains. These results are consistent with the idea that TCR-Vβ genes have been evolutionarily selected to react with MHC proteins^33^. It is currently unknown if the differential TCR-Vα usage by mature 6F/6F T-cells is primarily the result of selection from the pool of TCR α/β heterodimers initially expressed on DP thymocytes or is due to iterative sampling of TCRα chains through processive TCRα locus rearrangement. In any event, it is notable that the main consequence of reducing TCR signalling potential is skewing of the mature αβTCR repertoire through alternative TCRα chain usage rather than failure of positive selection.

Although it might have been predicted that significant differences in TCRα chain usage by positively selected αβTCRs would skew the potential antigen-reactive repertoire of the mature T-cell pool, we failed to identify any obvious ‘gaps’ in the TCR repertoire, which would be revealed by non-responsiveness to particular foreign antigens. Although by no means comprehensive, these results suggest that there is a striking degree of plasticity in the selection process that ensures preservation of the antigen-specific TCR repertoire despite attenuation of the TCR signalling potential. These findings raise the interesting possibility that, similar to ‘superantigen’ recognition, ligand recognition is primarily dictated by the TCRβ chain with TCRα functioning to stabilize TCRβ/pMHC interactions or adjust ligand-binding affinity^34^.

γδ T-cell and iNKT-cell development were impaired to a greater extent in 6F/6F mice than was αβ T-cell development. By definition, immature γδ T-cells and iNKT-cells, which express invariant or semi-invariant TCRs, are less prone to ‘revision’ of TCR–ligand binding affinity by pairing of TCRγ or TCRβ chains with alternate TCRδ and TCRα chains, respectively. Unlike conventional αβ T-cells, maturation of γδ T-cells and iNKT cells also apparently requires interaction with their cognate ligands in the thymus and is thought to be contingent on relatively high intensity TCR signalling^25^,^26^,^27^. Negative selection of conventional αβTCR^+^ thymocytes by clonal deletion, which also requires high intensity TCR signalling, was likewise impaired in 6F/6F mice. However, we also found that ‘clonal diversion’ of autoreactive thymocytes to the αβTCR^+^ DN fate is increased in 6F/6F mice, similar to the effect observed when CD28 co-receptor stimulation is removed or when pro-survival factors are overexpressed in DP thymocytes. The fact that thymocytes are still negatively selected, *albeit* by clonal diversion rather than clonal deletion, helps to explain the absence of autoimmune disease in 6F/6F mice^20^.

Similar to what we had observed in 6F/6F thymocytes^20^, the impact of inactivating CD3ζ ITAMs on TCR signalling responses varied considerably depending on the signalling pathway and the proximity of the downstream effectors to the TCR. TCR-mediated activation of proximal effectors including ZAP-70, SLP-76 and Cbl was reduced in 6F/6F T-cells, whereas the effect on downstream responses was variable. Activation of some intermediates such as Erk and Akt was strongly attenuated, whereas calcium mobilization and activation of NF-κB, Jnk and p38 were unimpaired or only mildly affected^20^. These results are most consistent with a kinetic proofreading model for TCR signalling where downstream effectors/pathways exhibit different activation thresholds, with some responses appearing to be more ‘analogue’ whereas others are more ‘digital’^35^. T-cell proliferation *in vitro* in response to TCR cross-linking or *in vivo* in response to LCMV infection was unaffected, slightly impaired, or in some cases enhanced in the absence of CD3ζ ITAMs. These results are concordant with those obtained when the cytosolic adapter SLP-76 was deleted or mutated to reduce TCR signalling potential which failed to observe an effect on T-cell proliferation^36^,^37^ but contrast markedly with those obtained using a different 6F/6F model system where it was found that a full complement of ITAMs is crucial for T-cell proliferation^19^. As discussed above, these discrepancies, which we believe can be attributed to differences in the model systems employed, further underscore the importance of retaining normal gene regulation and cell specificity in the design of mouse models of TCR signal attenuation.

In a previous study, we documented that the differential effects of ITAM reduction on downstream signalling pathways, most notably attenuation of Akt activation and relative sparing of the calcium and NF-κB pathways favors Treg development and results in an increase in the number of both thymus derived and peripherally derived Tregs in 6F/6F mice^20^. In this report, we show that the same reduction in the number of TCR ITAMs significantly impairs the generation of TFH cells *in vitro* in response to TCR cross-linking or in *in vivo* in response to antigenic stimulation and that this defect is T-cell intrinsic. It is worth noting that, similar to iNKT-cells and γδ T-cells, TFH cells have been shown to require high avidity TCR interactions and/or sustained TCR signalling for their development^32^,^38^. We found that in comparison to 6Y/6Y mice, induction of Bcl6, which is essential for the generation of TFH cells, was reduced in TFH cells in 6F/6F mice. In addition, we observed that IL-2 production in response to TCR engagement is not impaired in 6F/6F T-cells. Since IL-2 receptor signalling inhibits Bcl6 expression^39^, the lack of a defect in IL-2 production by 6F/6F T-cells along with attenuated TCR signalling potential provides an explanation for the reduced efficiency of TFH induction. It is also interesting to note that 45 days after LCMV infection, 6F/6F mice exhibited a higher T_EM_/T_CM_ ratio of peptide-specific CD4-SP and CD8-SP T-cells, a result consistent with recent data demonstrating that IL-2 and Bcl6 exert opposite effects on the induction of T_EM_ and T_CM_ cells^40^.

What insights do the current results provide regarding the ‘raison d'être’ for a multi-ITAM TCR signalling structure? The selective requirement for CD3ζ ITAMs, and by extension, ITAM-mediated signal amplification, for T-cell developmental and functional responses that are regulated by high avidity TCR/pMHC interactions suggests that it is important to regulate these events by linking them to high signalling thresholds. A high signalling threshold for triggering negative selection is logically consistent with the idea that this event should be tightly restricted to avoid limiting the potentially useful TCR repertoire and with the notion that high intensity signalling is coupled to initiation of specific cellular events that induce apoptosis^22^. Likewise, the threshold for TFH-cell induction is presumably intentionally set high (high affinity ligand binding, high signal intensity) to limit this event to ‘full blown’ antigen/pathogen responses, particularly as inadvertent TFH-cell induction by self-peptides might lead to severe autoimmunity. One way to adjust the activation threshold is through TCR signal amplification (mediated by multiple ITAMs) in response to high affinity/long dwell time interactions. According to this idea, only strong interactions result in strong signals through the activation (phosphorylation) of multiple TCR ITAMs. Notably, reduction of the TCR signalling potential does not appear to significantly restrict the antigen-reactive mature T-cell repertoire, nor does it substantially impact several ‘general’ T-cell responses, including proliferation and cytokine production. Thus, ITAM-mediated signal amplification is selectively required for and linked to specific effector responses. These findings contribute to the objective of accurately predicting the effects of TCR signal attenuation on both T-cell maturation and T-cell functional responses as a means of evaluating potential targeted and off-target effects of drug treatments for human immune disorders.

## Methods

### Animal models and statistics

The generation of 6Y/6Y and 6F/6F ‘knock-in’ mice was described previously^20^. Both mutant lines were back-crossed to C57BL/6 mice for at least 10 generations. H-Y TCR transgenic, AND TCR transgenic in the C57BL/6 background and B6:CD45.1 congenic mice were obtained from the NIAID Taconic exchange repository. Mice were housed in a barrier facility and all procedures in this study were approved by the NIH Animal Care and Use Committee (ACUC). All experiments were conducted with age-matched (typically 4–6 months old) and gender-matched control and experimental groups. In bar graphs, error bars are s.d. or s.e.m. as noted in the figure legend. Statistical analysis was performed with two-tailed, unpaired Student’s *t*-tests with GraphPad Prism software (For all figures: *P<0.05; **P<0.01; ***P<0.001). The number of mice used per experiment was determined by several factors: (1) The ACUC directive to use the minimum number of animals required to obtain valid results; (2) a minimum number of three mice per group for statistical confidence if individual samples could be analysed separately; (3) larger numbers of mice were used for pooling of cells from individuals if rare or purified cell populations were required for specific experiments; (4) in some cases, such as enumeration of thymocytes or splenocytes, samples were available and could be included from mice killed for other experiments. Any animals exhibiting signs of disease, wasting or failure to thrive were excluded from the study.

### Antibodies reagents and flow cytometery

Fluorescent dye-labelled antibodies to CD45.1(A20), CD4(RM4–5), CD5(53–7.3), CD8(53–6.7), CD24(ML5), CD25(PC61.5), CD44(1M7), TCRβ(H57–597), CD69(H1.2F3), CD62L(MEL-14), CXCR5(2G8), PD-1(J-43), CD95/Fas(Jo2), Bcl6(K112–91), Vα2(B20.1), Vα3.2(RR3–16), Vα8.3(B21.4), Vα11(RR8.1), TCRγδ(GL3), NK1.1(PK136), IL-17a(TC11–18H10), IFN-γ(554413)), TNF-α(MP6-XT22), IL-2(JES6-SH4) and the mouse TCRβ Screening panel (Cat#557004) were from BD Biosciences. Fluorescent dye-labelled antibodies to KLRG1(2F1), Foxp3(FKJ-16S), and Nur77(12.14) were purchased from eBioscience. Recombinant IL-2, IL-6 and TGF-β were from R&D Systems. Fluorochrome-conjugated PBS-57:CD1d and LCMV peptide tetramers (GP67–77, GP33–41, NP396–404, NP205–212 and GP276–286) were obtained from the NIH Tetramer Core Facility. Biotin-labelled soluble I-Ab molecules containing 2W, OVA, LLO190–201 or FliC427–441 peptides covalently attached to the I-Ab beta chain were produced with the I-Ab alpha chain in *Drosophila melanogaster* S2 cells, then purified and made into tetramers with streptavidin (SA)–phycoerythrin or SA–allophycocyanin (Prozyme, San Leandro, CA, USA) as described previously^30^. Cells were pre-incubated with blocking antibody (2.4G2) for 5 min then incubated for 15 min on ice with all specific antibodies. Samples were run on a FACSCaliber or LSRII cytometer (Becton Dickinson) and analysed with FlowJo software (TreeStar). For intracellular cytokine staining, cells were incubated with brefeldin A for the final 2 h of a 5-h re-stimulation with PMA (phorbol 12-myristate 13-acetate) plus ionomycin. Permeabilization kits from eBioscience and BD Biosciences were used for intracellular staining.

### Cell stimulation and Western blotting

Cell stimulation, calcium mobilization and Western blotting experiments were performed as described^20^. Briefly, lymph node T-cells (5 × 10^6^) were stimulated at 37 °C during the indicated periods of time with preformed immune complexes composed of biotinylated anti-CD3ε (10 mg ml^−1^; 145-2C11; BD Biosciences) and SA (30 mg ml^−1^; Sigma Aldrich). After stimulation, cells were immediately resuspended in lysis buffer containing: 1% Triton X-100, 10 mM Tris pH 7.4, 150 mM NaCl, 2 mM Na_3_VO_4_, 10 mM NaF, 1 mM EDTA and 1 protease inhibitor tablet (Roche). Cell equivalents (2 × 10^6^ cells) were loaded on 4–12% gradient gels (Invitrogen) and were analysed by immunoblot. Antibodies used for blotting included Notch1 (D1E11), Myc (9402), pErk (9101) and pAkt (9271), ZAP phosphorylated at Tyr319 (2701), from Cell Signaling Technologies; pSLP-76 (558367) from BD Bioscience; pCbl (26140) from Santa Cruz Biotechnologies For stimulation of T-cells after LCMV infection the following peptides were used: GP61–80, NP396–404, GP33–41, NP205–212 and GP276–286. Full size images are presented in Supplementary Fig. 3c,d.

### Cell purification and CFSE labelling

Purified T-cell populations were obtained from lymph nodes using a magnetic bead/column system (MACS; Miltenyi Biotech). Naïve CD4-SP T-Cells were isolated by first labelling total lymph node cells with anti-B220 biotin, anti-CD11b biotin, anti-CD8 biotin, anti-CD44 biotin and anti-CD25 biotin. CD4-SP cells (unlabelled fraction) were isolated by magnetic columns after being washed and labelled with strepavidin microbeads. Purity was >90%. Tetramer-mediated cell enrichment was performed as described^30^. For CFSE (5-(and -6)-carboxyfluorescein diacetate succinimidyl ester, Molecular Probes) labelling, cells were incubated with 5μg ml^−1^ of CFSE for 15 min at 37 °C. Cell division was assessed by determining the percentage of CFSE^lo/med^ cells by flow cytometry and dividing by the percentage of CFSE^lo/med^ cells obtained following stimulation with PMA and ionomycin.

### Virus infections and immunizations

Eight to 12 week-old gender-matched 6Y/6Y and 6F/6F mice were infected intravenously with 2 × 10^6^ PFU of LCMV Armstrong clone 53b. Stocks were prepared by a single passage on BHK-21 cells, and viral titres were determined by plaque formation on Vero cells. For *in vivo* TFH induction, mice were injected intraperitoneally with SRBCs (5 × 10^8^; Innovative Research) or NP-CGG, NP-KLH (100 μg; Vector Labs). IgM and IgG enzyme-linked immunosorbent assays (BD Biosciences) were performed with NP4-BSA- or NP32-BSA-coated plates (Vector Labs).

### Generation of bone marrow chimeras

Donor bone marrow cells were depleted of lineage-positive cells by the addition of anti-CD3, anti-CD4, anti-CD8, anti-CD11b, anti-Ter119 and anti-CD19 antibodies followed by purification using the magnetic column system. About 5 × 10^6^ cells were injected into the tail veins or retro-orbital sinus of irradiated B6 (950 rads) mice.

### Immunohistochemistry

Mouse tissues were fixed in 4% paraformaldehyde (Protocol) and embedded in paraffin. Sections (6 μm) were stained with haematoxylin and eosin, or, for immunofluorescence staining, tissue sections were fixed in 4% (wt/vol) paraformaldehyde and blocked with 1% (wt/vol) BSA and 10% (vol/vol) goat serum in PBS before incubation with fluorescein isothiocyanate-conjugated rat anti-mouse B220 (abCAM) or rat anti-mouse CD3 (abCAM). Images were obtained on an Axiovert LSM 510-META inverted microscope (Zeiss), except for tiled images, which were captured and assembled on a Fluoview FV1000 (Olympus).

### Real-time PCR

For gene-expression studies, total cell RNA was isolated with a PicoPure RNA Isolation kit (Arcturus). RNA samples (100 ng of each) were reverse transcribed with the SuperScript First-Strand Synthesis system (Invitrogen) and were assayed by RT-PCR. Transcripts were quantified with a Roche LightCycler 480. Duplicates were run for each sample in a 96-well plate; *Actb* (encoding b-actin) served as the endogenous reference gene. The relative quantification method was used, with the ratio of the messenger RNA (mRNA) abundance of the gene of interest normalized to the abundance of β-actin mRNA and with the average of control thymocyte samples serving as the calibrator value. The specificity of the products was confirmed by melting curves and electrophoresis.

### TCR-Vβ sequencing

Naïve (CD44^−^CD25^−^) CD4-SP T-cells were purified from 8-week-old female 6Y/6Y and 6F/6F mice and genomic DNA was extracted using a Qiagen DNA isolation kit. Deep sequencing of Vβ CDR3 regions was performed by Adaptive Biotechnologies, Seattle, WA, USA.

## Additional information

**How to cite this article:** Hwang, S. *et al*. TCR ITAM multiplicity is required for the generation of follicular helper T-cells. *Nat. Commun*. 6:6982 doi: 10.1038/ncomms7982 (2015).

## Supplementary Material

Supplementary Information Supplementary Figures 1-8

## References

[b1] Manolios N., Letourneur F., Bonifacino J. S. & Klausner R. D. Pairwise, cooperative and inhibitory interactions describe the assembly and probable structure of the T-cell antigen receptor. EMBO J. 10, 1643–1651 (1991). 1828760 10.1002/j.1460-2075.1991.tb07687.x PMC452834

[b2] Call M. E., Pyrdol J. & Wucherpfennig K. W. Stoichiometry of the T-cell receptor-CD3 complex and key intermediates assembled in the endoplasmic reticulum. EMBO J. 23, 2348–2357 (2004). 15152191 10.1038/sj.emboj.7600245 PMC423287

[b3] Reth M. Antigen receptor tail clue. Nature 338, 383–384 (1989). 2927501 10.1038/338383b0

[b4] Flaswinkel H., Barner M. & Reth M. The tyrosine activation motif as a target of protein tyrosine kinases and SH2 domains. Semin. Immunol. 7, 21–27 (1995). 7612891 10.1016/1044-5323(95)90004-7

[b5] Aivazian D. & Stern L. J. Phosphorylation of T cell receptor zeta is regulated by a lipid dependent folding transition. Nat. Struct. Biol. 7, 1023–1026 (2000). 11062556 10.1038/80930

[b6] Smith-Garvin J. E., Koretzky G. A. & Jordan M. S. T cell activation. Annu. Rev. Immunol. 27, 591–619 (2009). 19132916 10.1146/annurev.immunol.021908.132706 PMC2740335

[b7] Neumeister E. N. . Binding of ZAP-70 to phosphorylated T-cell receptor zeta and eta enhances its autophosphorylation and generates specific binding sites for SH2 domain-containing proteins. Mol. Cell Biol. 15, 3171–3178 (1995). 7760813 10.1128/mcb.15.6.3171 PMC230549

[b8] Tailor P. . The proline-rich sequence of CD3epsilon as an amplifier of low-avidity TCR signalling. J. Immunol. 181, 243–255 (2008). 18566390 10.4049/jimmunol.181.1.243 PMC2665931

[b9] Deford-Watts L. M. . The cytoplasmic tail of the T cell receptor CD3 epsilon subunit contains a phospholipid-binding motif that regulates T cell functions. J. Immunol. 183, 1055–1064 (2009). 19542373 10.4049/jimmunol.0900404 PMC2954055

[b10] Ravichandran K. S. . Interaction of Shc with the zeta chain of the T cell receptor upon T cell activation. Science 262, 902–905 (1993). 8235613 10.1126/science.8235613

[b11] Love P. E. & Shores E. W. ITAM multiplicity and thymocyte selection: how low can you go? Immunity 12, 591–597 (2000). 10894159 10.1016/s1074-7613(00)80210-1

[b12] Shores E. W. . Role of the multiple T cell receptor (TCR)-zeta chain signalling motifs in selection of the T-cell repertoire. J. Exp. Med. 185, 893–900 (1997). 9120395 10.1084/jem.185.5.893 PMC2196173

[b13] van Oers N. S., Love P. E., Shores E. W. & Weiss A. Regulation of TCR signal transduction in murine thymocytes by multiple TCR zeta-chain signalling motifs. J. Immunol. 160, 163–170 (1998). 9551968

[b14] Shores E. W. . Role of TCR zeta chain in T-cell development and selection. Science 266, 1047–1050 (1994). 7526464 10.1126/science.7526464

[b15] Sommers C. L. . Function of CD3 epsilon-mediated signals in T-cell development. J. Exp. Med. 192, 913–919 (2000). 10993922 10.1084/jem.192.6.913 PMC2193290

[b16] Haks M. C. . A redundant role of the CD3 gamma-immunoreceptor tyrosine-based activation motif in mature T cell function. J. Immunol. 166, 2576–2588 (2001). 11160319 10.4049/jimmunol.166.4.2576

[b17] Pitcher L. A. . The CD3 gamma epsilon/delta epsilon signalling module provides normal T cell functions in the absence of the TCR zeta immunoreceptor tyrosine-based activation motifs. Eur. J. Immunol. 35, 3643–3654 (2005). 16259006 10.1002/eji.200535136

[b18] Holst J. . Scalable signalling mediated by T cell antigen receptor-CD3 ITAMs ensures effective negative selection and prevents autoimmunity. Nat. Immunol. 9, 658–666 (2008). 18469818 10.1038/ni.1611

[b19] Guy C. S. . Distinct TCR signalling pathways drive proliferation and cytokine production in T cells. Nat. Immunol. 14, 262–270 (2013). 23377202 10.1038/ni.2538 PMC3577985

[b20] Hwang S. . Reduced TCR signalling potential impairs negative selection but does not result in autoimmune disease. J. Exp. Med. 209, 1781–1795 (2012). 22945921 10.1084/jem.20120058 PMC3457736

[b21] Mariathasan S., Jones R. G. & Ohashi P. S. Signals involved in thymocyte positive and negative selection. Semin. Immunol. 11, 263–272 (1999). 10441212 10.1006/smim.1999.0182

[b22] Starr T. K., Jameson S. C. & Hogquist K. A. Positive and negative selection of T cells. Annu. Rev. Immunol. 21, 139–176 (2003). 12414722 10.1146/annurev.immunol.21.120601.141107

[b23] Pobezinsky L. A. . Clonal deletion and the fate of autoreactive thymocytes that survive negative selection. Nat. Immunol. 13, 569–578 (2012). 22544394 10.1038/ni.2292 PMC3362677

[b24] Hayes S. M. & Love P. E. Strength of signal: a fundamental mechanism for cell fate specification. Immunol. Rev. 209, 170–175 (2006). 16448542 10.1111/j.0105-2896.2006.00356.x

[b25] Hayes S. M., Li L. & Love P. E. TCR signal strength influences alphabeta/gammadelta lineage fate. Immunity 22, 583–593 (2005). 15894276 10.1016/j.immuni.2005.03.014

[b26] Haks M. C. . Attenuation of gammadeltaTCR signalling efficiently diverts thymocytes to the alphabeta lineage. Immunity 22, 595–606 (2005). 15894277 10.1016/j.immuni.2005.04.003

[b27] Bendelac A., Savage P. B. & Teyton L. The biology of NKT cells. Annu. Rev. Immunol. 25, 297–336 (2007). 17150027 10.1146/annurev.immunol.25.022106.141711

[b28] Becker A. M. . Invariant NKT-cell development requires a full complement of functional CD3 zeta immunoreceptor tyrosine-based activation motifs. J. Immunol. 184, 6822–6832 (2010). 20483726 10.4049/jimmunol.0902058 PMC2947369

[b29] Dao T. . Development of CD1d-restricted NKT cells in the mouse thymus. Eur. J. Immunol. 34, 3542–3552 (2004). 15549774 10.1002/eji.200425546

[b30] Moon J. J. . Naive CD4(+) T cell frequency varies for different epitopes and predicts repertoire diversity and response magnitude. Immunity 27, 203–213 (2007). 17707129 10.1016/j.immuni.2007.07.007 PMC2200089

[b31] Joshi N. S. . Inflammation directs memory precursor and short-lived effector CD8(+) T cell fates via the graded expression of T-bet transcription factor. Immunity 27, 281–295 (2007). 17723218 10.1016/j.immuni.2007.07.010 PMC2034442

[b32] Fazilleau N., McHeyzer-Williams L. J., Rosen H. & McHeyzer-Williams M. G. The function of follicular helper T cells is regulated by the strength of T cell antigen receptor binding. Nat. Immunol. 10, 375–384 (2009). 19252493 10.1038/ni.1704 PMC2712297

[b33] Yin L., Scott-Browne J., Kappler J. W., Gapin L. & Marrack P. T cells and their eons-old obsession with MHC. Immunol. Rev. 250, 49–60 (2012). 23046122 10.1111/imr.12004 PMC3963424

[b34] Andersen P. S. . Role of the T cell receptor alpha chain in stabilizing TCR-superantigen-MHC class II complexes. Immunity 10, 473–483 (1999). 10229190 10.1016/s1074-7613(00)80047-3

[b35] Lever M., Maini P. K., van der Merwe P. A. & Dushek O. Phenotypic models of T cell activation. Nat. Rev. Immunol. 14, 619–629 (2014). 25145757 10.1038/nri3728

[b36] Smith-Garvin J. E. . T-cell receptor signals direct the composition and function of the memory CD8+ T-cell pool. Blood 116, 5548–5559 (2010). 20847203 10.1182/blood-2010-06-292748 PMC3031403

[b37] Wiehagen K. R. . Loss of tonic T-cell receptor signals alters the generation but not the persistence of CD8+ memory T cells. Blood 116, 5560–5570 (2010). 20884806 10.1182/blood-2010-06-292458 PMC3031404

[b38] Tubo N. J. . Single naive CD4+ T cells from a diverse repertoire produce different effector cell types during infection. Cell 153, 785–796 (2013). 23663778 10.1016/j.cell.2013.04.007 PMC3766899

[b39] Johnston R. J., Choi Y. S., Diamond J. A., Yang J. A. & Crotty S. STAT5 is a potent negative regulator of TFH cell differentiation. J. Exp. Med. 209, 243–250 (2012). 22271576 10.1084/jem.20111174 PMC3281266

[b40] Pepper M., Pagan A. J., Igyarto B. Z., Taylor J. J. & Jenkins M. K. Opposing signals from the Bcl6 transcription factor and the interleukin-2 receptor generate T helper 1 central and effector memory cells. Immunity 35, 583–595 (2011). 22018468 10.1016/j.immuni.2011.09.009 PMC3208313

