# Supplementary material for: TCR ITAM multiplicity is required for the generation of follicular helper T-cells
Source: Nat Commun. 2015 May 11;6:6982. doi: 10.1038/ncomms7982 (PMC4428620; doi:10.1038/ncomms7982)
Supplement: Supplementary Information — Supplementary Figures 1-8 [file ncomms7982-s1.pdf]

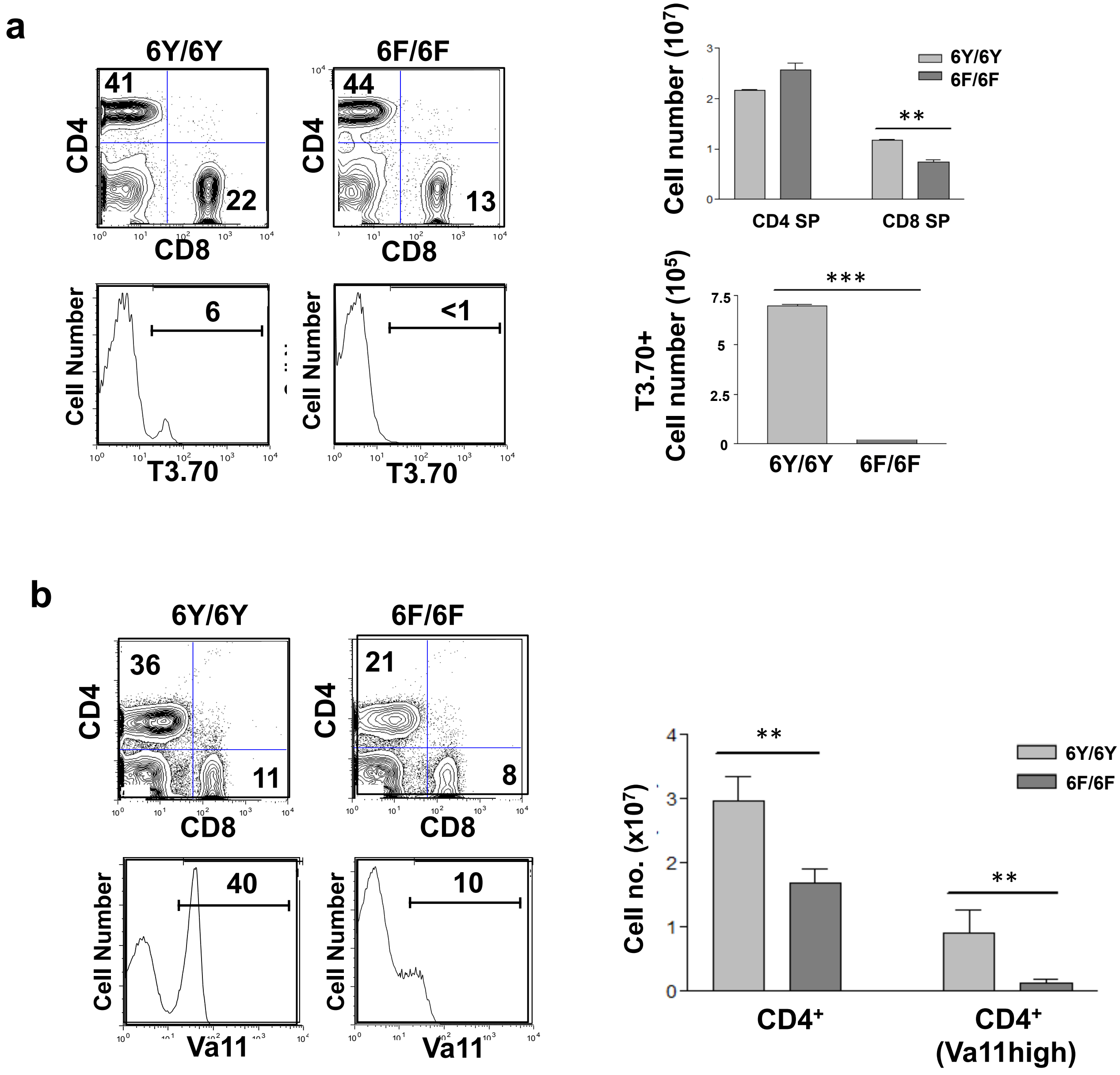

Supplementary Figure 1. T cell development in TCR transgenic 6F/6F mice. (a) Upper panels, CD4 vs CD8 profile of lymph node cells from H-Y;6Y/6Y and H-Y; 6F/6F female mice. Lower panels, staining of total lymph node cells with antibody to H-Y TCR (T3.70). Right upper panel, number of CD4-SP and CD8-SP splenocytes in H-Y;6Y/6Y and H-Y; 6F/6F female mice. Right lower panel, number of T3.70^{+} CD8-SP splenocytes in H-Y;6Y/ 6Y and H-Y; 6F/6F female mice. Data are from 10 mice of each genotype. (b) Upper panels, CD4 vs CD8 profile of lymph node cells from AND;6Y/6Y and AND; 6F/6F female mice. Lower panels, staining of total lymph node cells with antibody to AND TCR Vα chain (Va11). Right, number of total CD4-SP splenocytes and Va11^{+} CD4-SP splenocytes in AND;6Y/6Y and AND; 6F/6F female mice. Data are from 10 mice of each genotype. For bar graphs, data were analyzed by Unpaired t-test (Two-tailed) and are represented as mean ± SD *p<.05; **p<.01; ***p<.005.

a **b**

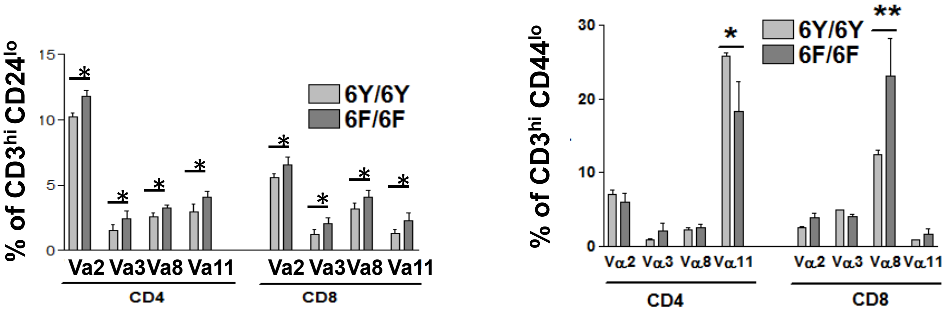

c

Sample Name	Total	Unique	Productive Total	Productive Uniques	Out of Frame Total	Out of Frame Uniques	Has Stop Total	Has Stop Uniques	Entropy	Clonality
6Y_naive	7453800	217462	5388941	144120	2021449	71699	43410	1643	16.44	0.041
6F_naive	8593203	231146	6445197	158092	2111574	71485	36432	1569	16.52	0.043
B6_naive	6261483	194645	4555565	129230	1671792	63986	34126	1429	16.26	0.043

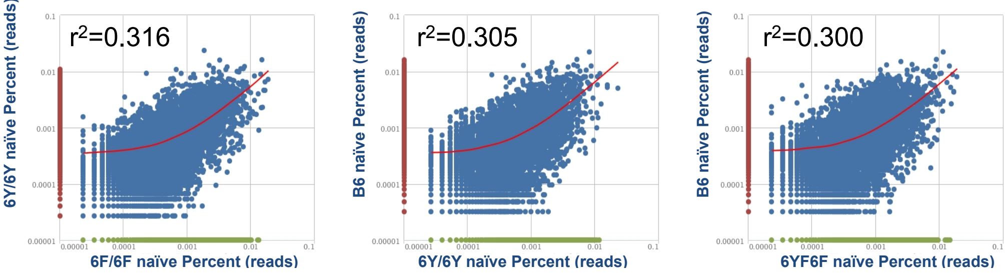

Adjusted Diversity*:	6Y/6Y	6F/6F	B6
	**196324**	**196921**	**191466**

*Adjusted diversity=measured diversity x square root of 10^6 /number of productive sequences.

d

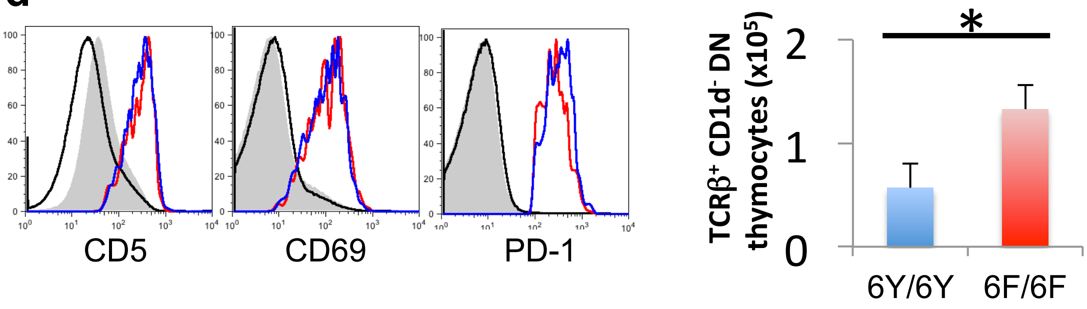

Supplementary Figure 2. TCRVα usage and TCRVβ sequencing of TCRs in 6F/6F mice. (a) TCR Vα usage in 6Y/6Y and 6F/6F mice. Thymocytes were stained with anti-CD3/4/8/24 and the indicated Vα antibodies. Shown are percentage of $CD3^{hi}$ $CD24^{lo}$ CD4-SP or CD8-SP thymocytes that express the indicated Vα chain. N = 5 mice of each genotype. (b) Vα usage in Vβ3 TCR transgenic 6Y/6Y and 6F/6F mice. Thymocytes (upper panel) or splenocytes (lower panel) were stained with anti-CD3/4/8/24 (thymocytes) or anti-CD3/4/8/44 (splenocytes) and the indicated Vα antibodies. Shown are percentage of $CD3^{hi}$ $CD24^{lo}$ CD4-SP or CD8-SP thymocytes or $CD3^{hi}$ $CD44^{lo}$ CD4-SP or CD8-SP splenocytes that express the indicated Vα chain. N = 6 mice of each genotype. (c) Upper panel, summary of results of deep sequencing of TCRβ chain V regions from naïve CD4-SP T cells from 6Y/6Y and 6F/6F mice. Lower panels, comparison plots of CDR3 DNA sequences data from B6, 6Y/6Y and 6F/6F CD4-T cells. Linear regression plots are shown in red. r^2 values are shown in each plot. (d) Enumeration of 'clonally diverted' DN thymocytes. Left panels, staining of DN $αβTCR^+$ PBS-57:CD1d-tetramer-negative 6Y/6Y (blue) or 6F/6F (red) thymocytes with CD5, CD69, PD-1. Staining of CD4+CD8+ thymocyes from 6Y/6Y (gray) or 6F/6F (blcak) mice are shown for comparison. Right panel, number of DN $αβTCR^+$ PBS-57:CD1d-tetramer-negative thymocytes in 6Y/6Y and 6F/6F mice. N = 3 mice of each genotype. For bar graphs, data are represented as mean ± SD *p<.05; **p<.01; ***p<.005.

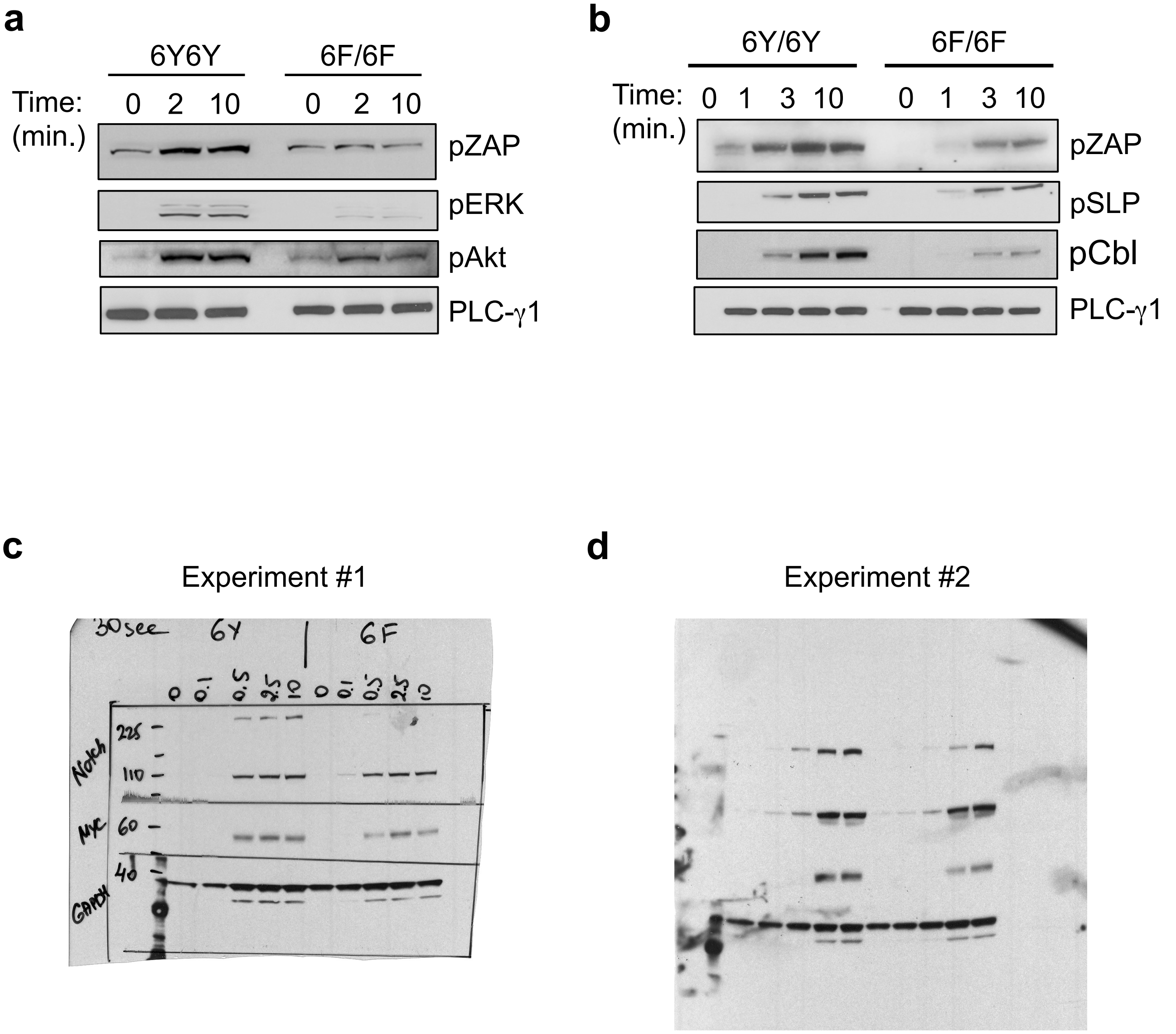

Supplementary Figure 3. TCR-mediated signaling in 6Y/6Y and 6F/6F T cells. (a,b) Naïve ($CD44^-$ $CD25^-$) lymph node CD4-SP T cells from 6Y/6Y and 6F/6F were stimulated for the indicated times with plate-bound anti-TCRβ + anti-CD28 and whole cell extracts were analyzed by SDS-PAGE and western blotting with the indicated antibodies. Results are representative of 3 independent experiments. (c) Original blots for data shown in Figure 3b.

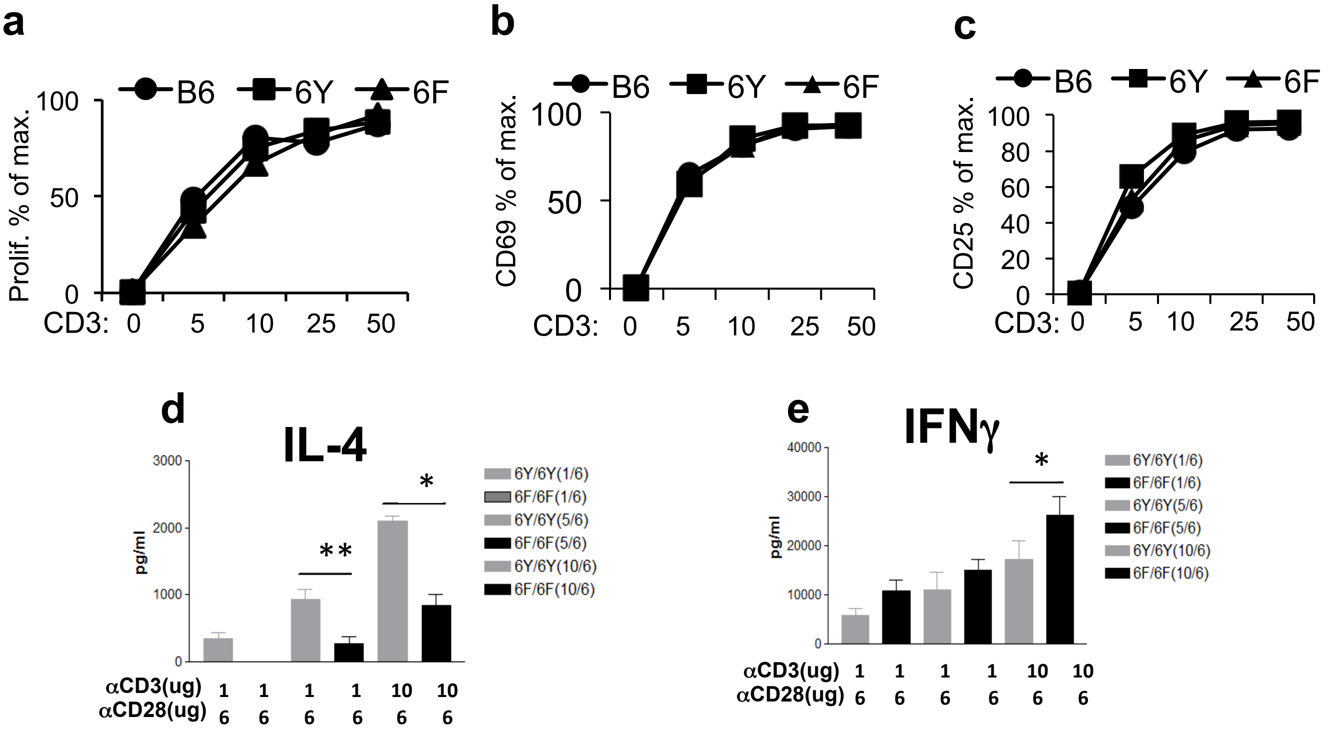

Supplementary Figure 4. Analysis of mature T cell function in 6Y/6Y and 6F/6F mice. (a) Proliferation of CD8-SP T cells. Naïve ($CD44^{lo}$) CD8-SP lymph node T cells were stimulated for 3 days with the indicated amount of plate-bound anti-CD3 antibody. (b,c) Activation-induced up-regulation of CD69 (b) and CD25 (c). Naïve ($CD44^{lo}CD25^{-}$) CD4-SP lymph node T cells were stimulated for 3 days with the indicated amount of plate-bound anti-CD3 antibody. Results are shown as percent of maximum proliferation with PMA + ionomycin as assessed by CFSE dilution. One representative of 3 independent experiments. (d,e) IL-4 and INFγ production by 6Y/6Y and 6F/6F T cells. Naïve CD4-SP T cells were incubated on antibody coated plates for 4 days under Th2 conditions or under Th1 conditions. Cells were rested overnight then stimulated for 4 hrs with PMA + ionomycin in the presence of golgi block, surface stained for CD4 and stained intracellularly for IL-4 or IFNγ. One representative of 2 experiments. Statistical data were analyzed by Unpaired t-test (Two-tailed) and are represented as mean ± SD. *p<.05; **p<.01.

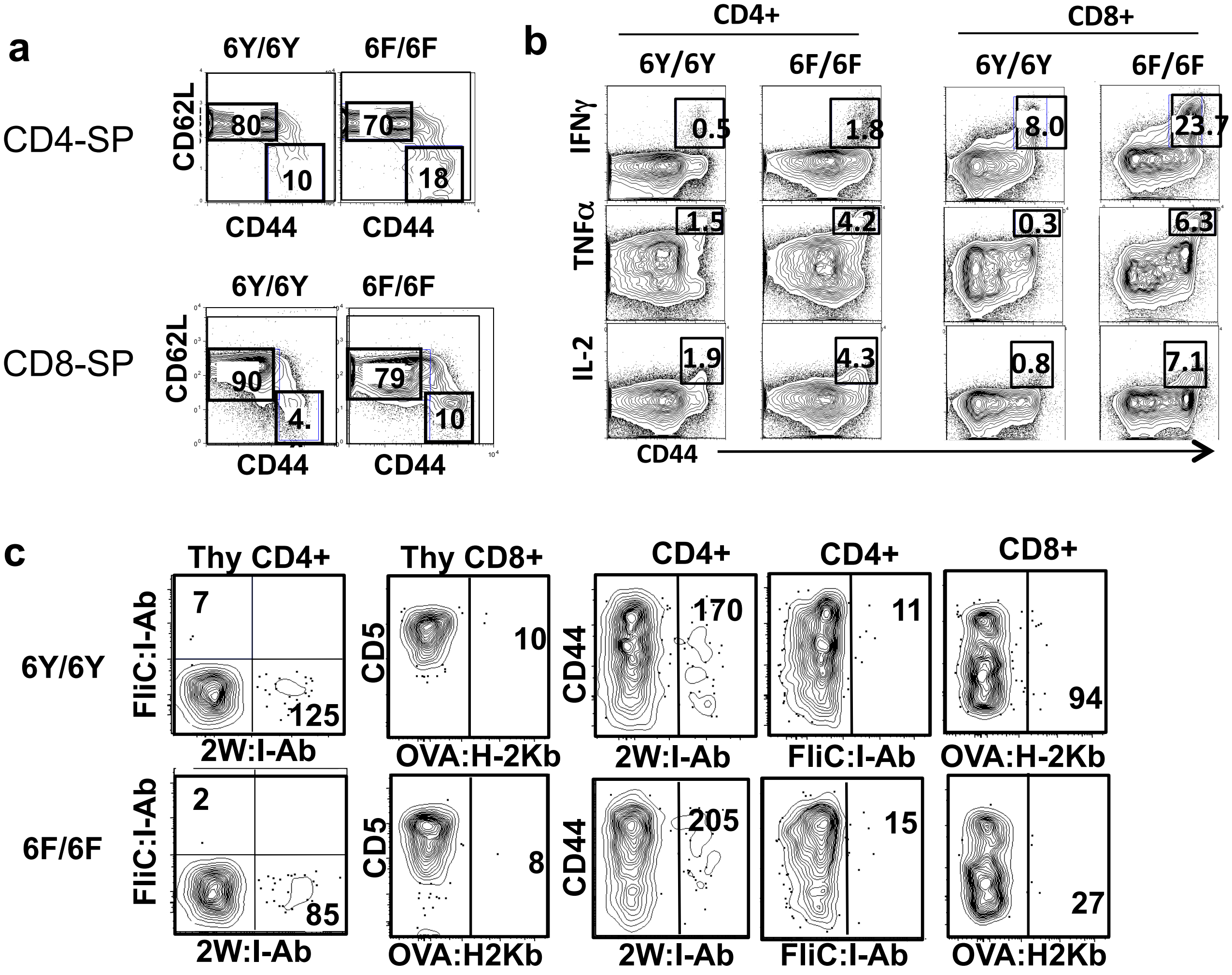

Supplementary Figure 5. Phenotype and naïve repertoire analysis of peripheral T cells in 6Y/6Y and 6F/6F mice. (a) CD44 vs CD62L staining profiles of gated CD4-SP (upper panel or CD8-SP (lower panel) lymph node T cells from 6Y/6Y or 6F/6F mice. Percentage of naïve ($CD62L^{hi}CD44^{lo}$) and memory phenotype ($CD62L^{lo}CD44^{hi}$) cells are shown. One representative of 8 experiments. (b) Percentage of cytokine positive T cells in 6Y/6Y and 6F/6F mice. Lymph node T cells were isolated from 6Y/6Y and 6F/6F mice, stimulated for 4 hrs with PMA + ionomycin in the presence of golgi block, surface stained for CD4/8/44 and stained intracellularly for IL-2, TNFα or IFNγ. Percentage of cytokine positive cells in the indicated gate is shown. Results shown are representative of 3 independent experiments. (c) Enumeration of antigen-specific T cell pools in un-immunized 6Y/6Y and 6F/6F mice. Total numbers of 2W/I-A^b specific, FliC/I-A^b specific or OVA/K^b specific thymocytes (2 left panels) or peripheral (lymph node and spleen) T cells (3 right panels) were analyzed by tetramer enrichment.

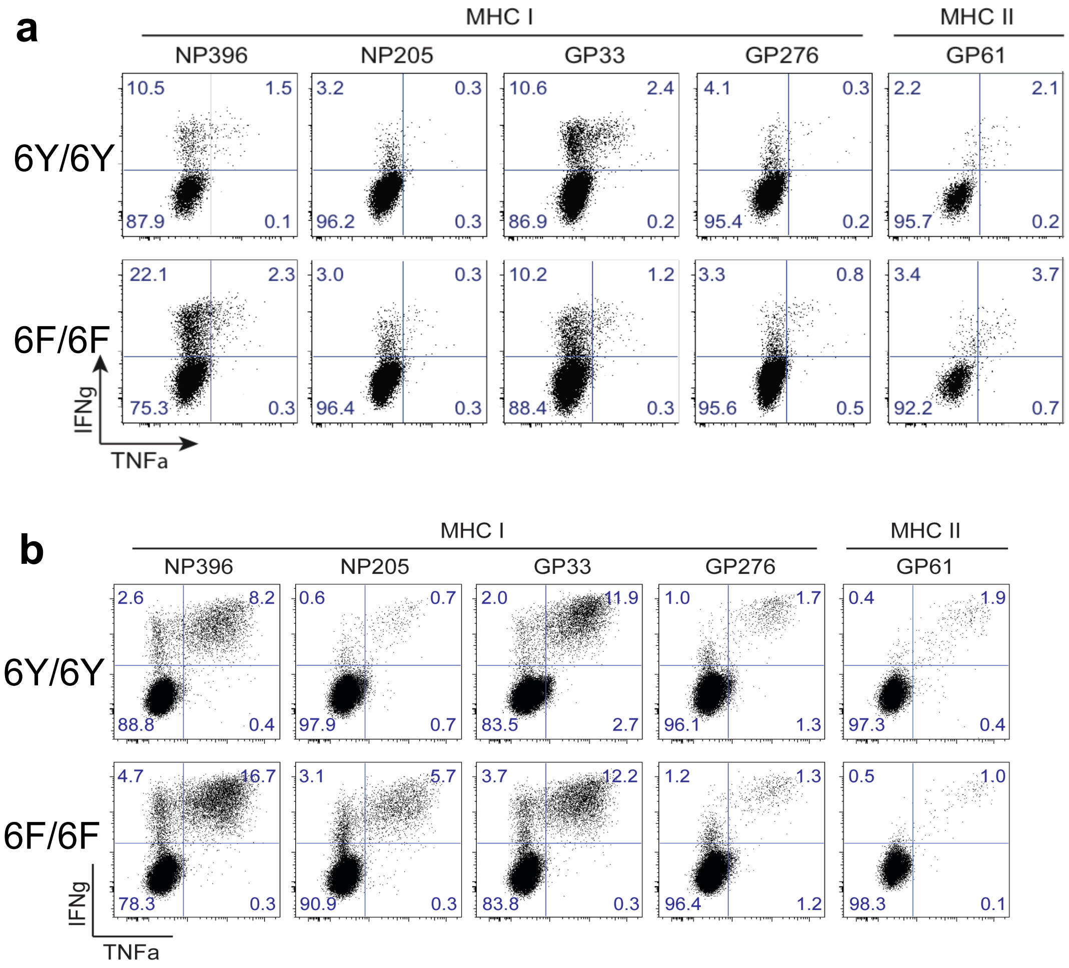

Supplementary Figure 6. T cell responses to LCMV infection.
Mice were infected with LCMV Armstrong and spleen T cells were analyzed 8 days (a) or 45 days (b) after infection. Plots show percentage of IFNγ^+ and/or TNFα^+ splenocytes detected after 5hr *in vitro* stimulation with the indicated peptides.

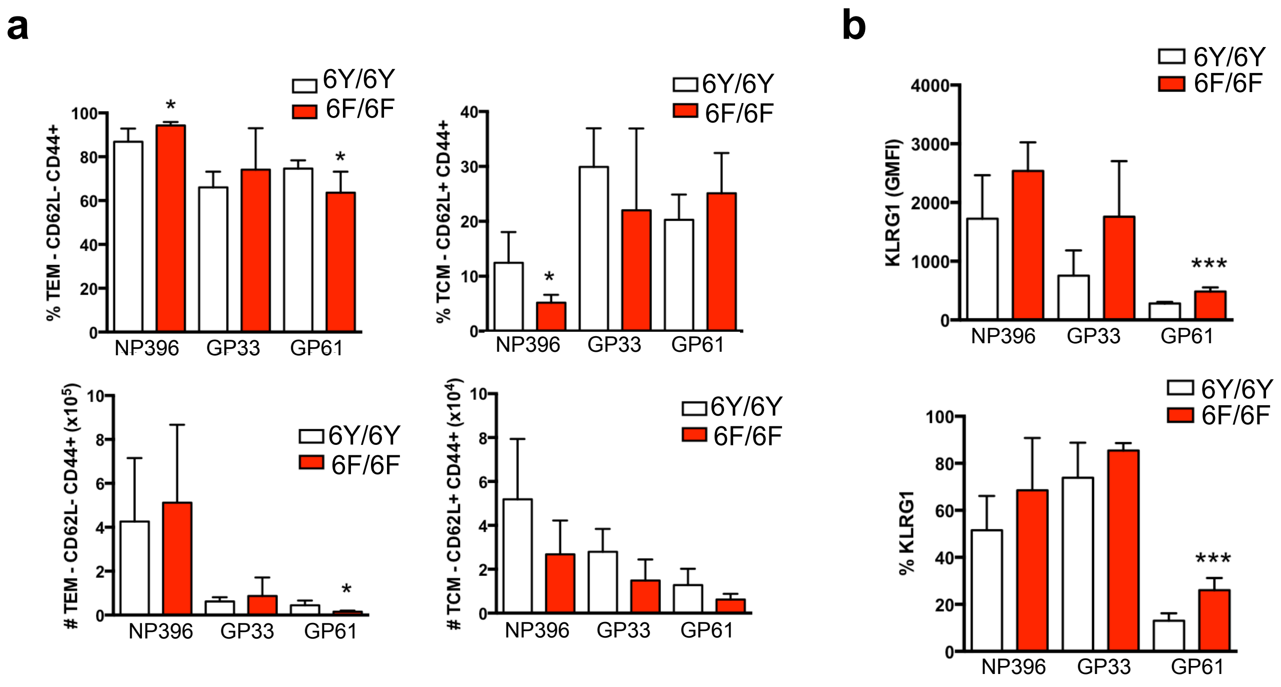

Supplementary Figure 7. Memory T cell responses to LCMV infection.
(a) Right, percentage (upper panel) or number (lower panel) of T effector memory phenotype tetramer-positive splenocytes 45 days after LCMV infection. Left, percentage (upper panel) or number (lower panel) of T central memory phenotype tetramer-positive splenocytes 45 days after LCMV infection. 6 mice were analyzed for each group. (b) MFI of KLRG1 surface staining (upper panel) or % KLRG1 positive cells (lower panel) bound by the indicated tetramer 45 days after LCMV infection. For bar graphs, data were analyzed by Unpaired t-test (Two-tailed**)** and are represented as mean ± SEM *p<.05; **p<.01; ***p<.005.

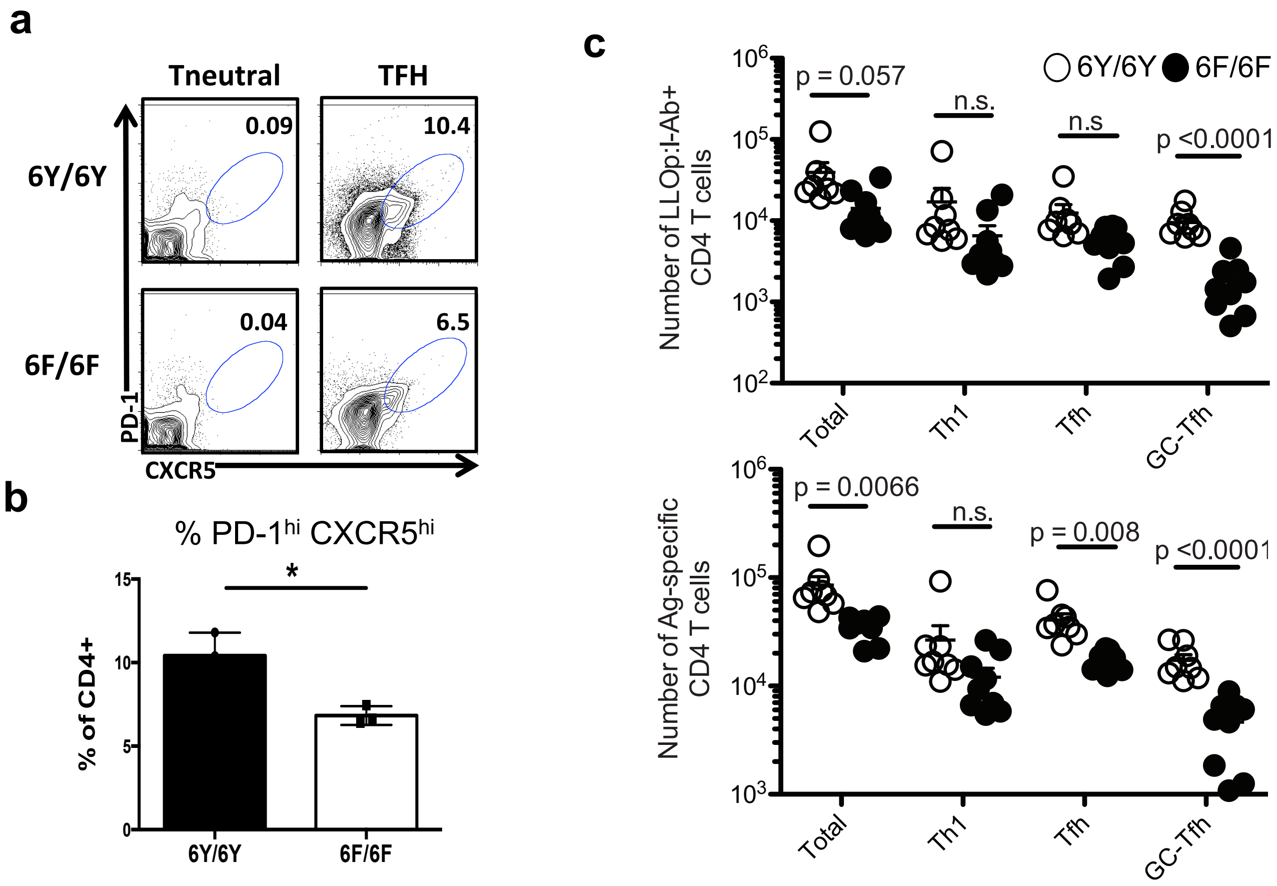

Supplementary Figure 8. TFH cell induction in 6Y/6Y and 6F/6F mice infected with *Listeria monocytogenes*. (a) *In vitro* induction of TFH from purified naïve ($CD44^-$ $CD25^-$) CD4-SP T cells from 6Y/6Y and 6F/6F mice. Shown are percentages of $CXCR5^+$ $PD\text{-}1^+$ TFH phenotype cells. (b) Summary of *in vitro* TFH induction experiments. N=3 mice of each genotype. (c) Age and gender-matched 6Y/6Y and 6F/6F mice (14 of each) were infected with *Listeria monocytogenes* that expresses 2W peptide and splenocytes were analyzed by FACS 10 days after infection. The number of Listeriolysin O (LLO)-A^b specific splenocytes was determined by tetramer staining. Th1 ($CD4^+IFN\gamma^+$), TFH ($CD4^+CXCR5^+PD\text{-}1^+$), GC-TFH ($CD4^+CXCR5^{hi}$ $PD\text{-}1^{hi}$). For bar graphs, data are represented as mean ± SD *p<.05; **p<.01; ***p<.005.
